# Dehydroascorbate induces plant resistance in rice against root‐knot nematode *Meloidogyne graminicola*

**DOI:** 10.1111/mpp.13230

**Published:** 2022-05-19

**Authors:** Satish Namdeo Chavan, Jonas De Kesel, Willem Desmedt, Eva Degroote, Richard Raj Singh, Giang Thu Nguyen, Kristof Demeestere, Tim De Meyer, Tina Kyndt

**Affiliations:** ^1^ Department of Biotechnology, Faculty of Bioscience Engineering Ghent University Ghent Belgium; ^2^ ICAR – Indian Institute of Rice Research Hyderabad India; ^3^ Department Plants and Crops, Faculty of Bioscience Engineering Ghent University Ghent Belgium; ^4^ Department of Green Chemistry and Technology, Faculty of Bioscience Engineering Ghent University Ghent Belgium; ^5^ Department of Data Analysis and Mathematical Modelling Ghent University Ghent Belgium

**Keywords:** dehydroascorbate, hydrogen peroxide (H_2_O_2_), induced resistance, *Meloidogyne graminicola*, rice, salicylic acid

## Abstract

Ascorbic acid (AsA) is an important antioxidant in plants and regulates various physiological processes. In this study, we show that exogenous treatments with the oxidized form of AsA, that is, dehydroascorbate (DHA), activates induced systemic resistance in rice against the root‐knot nematode *Meloidogyne graminicola*, and investigate the molecular and biochemical mechanisms underlying this phenotype. Detailed transcriptome analysis on roots of rice plants showed an early and robust transcriptional response on foliar DHA treatment, with induction of several genes related to plant stress responses, immunity, antioxidant activity, and secondary metabolism already at 1 day after treatment. Quantitative and qualitative evaluation of H_2_O_2_ levels confirmed the appearance of a reactive oxygen species (ROS) burst on DHA treatment, both at the site of treatment and systemically. Experiments using chemical ROS inhibitors or scavengers confirmed that H_2_O_2_ accumulation contributes to DHA‐based induced resistance. Furthermore, hormone measurements in DHA‐treated plants showed a significant systemic accumulation of the defence hormone salicylic acid (SA). The role of the SA pathway in DHA‐based induced resistance was confirmed by nematode infection experiments using an SA‐signalling deficient *WRKY45*‐RNAi line and reverse transcription‐quantitative PCR on SA marker genes. Our results collectively reveal that DHA activates induced systemic resistance in rice against the root‐knot nematode *M. graminicola*, mediated through the production of ROS and activation of the SA pathway.

## Graphical abstract

Dehydroascorbate treatment activates systemic induced resistance in rice against root‐knot nematode *Meloidogyne graminicola*, mediated via reactive oxygen species and the salicylic acid signalling pathway.

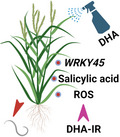

## 1 INTRODUCTION

Rice provides the staple food for more than half of the world's population (Beighley, 2010; FAO, 2021). The root‐knot nematode (RKN) *Meloidogyne graminicola* is one of the most important plant‐parasitic nematodes affecting rice production (Mantelin et al., 2017; Prasad et al., 2010; Ravindra et al., 2017) and is present in most rice‐growing areas globally (Dutta et al., 2012; Mantelin et al., 2017). The threat posed by *M. graminicola* is growing as traditional flooded rice systems are increasingly replaced by aerobic rice systems that are more water‐efficient and ecofriendly but also more amenable to RKN infestation (Mantelin et al., 2017; Ravindra et al., 2017). The second‐stage juveniles of *M. graminicola* can survive and remain viable in soil without a host plant for up to 5 months (Soomro, 1989). The control of RKNs using conventional methods is challenging because of its broad host range, ability to survive in soil, and the lack of a strong resistance source in elite rice cultivars (Bridge et al., 2005; Mantelin et al., 2017; Prasad et al., 2010). Induced resistance (IR) is one of the promising approaches in the search for environmentally‐friendly crop protection methods (Martínez‐Medina et al., 2017; Van Aubel et al., 2014; Walters & Fountaine, 2009).

IR refers to a state of reduced disease susceptibility of a plant induced by exposure to an external stimulus (De Kesel et al., 2021). Examples of IR stimulants include beneficial microbes such as *Trichoderma* spp. (Martínez‐Medina et al., 2017), natural compounds like piperonylic acid (Desmedt et al., 2021) and thiamine (Huang et al., 2016), and chemical compounds like the salicylate homologue acibenzolar‐S‐methyl (Romero et al., 2001). IR involves both the activation of direct defence responses, where defence pathways are induced, locally or systemically, on contact with the IR stimulus (De Kesel et al., 2021), and the so‐called (defence) priming phenomenon, where defence responses are more potently activated on subsequent challenge by stress (Conrath et al., 2006). Treatment of plants with IR stimulants leads to local and systemic transcriptional reprogramming and physiological changes (Desmedt et al., 2021; Mauch‐Mani et al., 2017). A variety of cellular responses have been reported to contribute to the IR phenotype, including alterations in ion transport across the plasma membrane, synthesis and secretion of secondary metabolites, accumulation of cell wall‐bound phenolics and lignin‐like polymers, callose deposition, activation of pathogenesis‐related (PR) genes, and reactive oxygen species (ROS) signalling (Conrath, 2009).

The oxidative burst—rapid accumulation of ROS, including superoxide radicals, hydrogen peroxide (H_2_O_2_), and hydroxyl radicals—is a primary feature of plant stress responses (Sharma et al., 2012; Wojtaszek, 1997). ROS are toxic to many organisms, but also act as signals in the induction of defence genes (Kuźniak & Urbanek, 2000). To be used as signalling molecules, ROS must be maintained at nontoxic levels through delicate balancing between generating and scavenging pathways (Deng et al., 2016). The antioxidant system that regulates H_2_O_2_ levels consists of enzymatic and nonenzymatic H_2_O_2_ scavengers (Niu & Liao, 2016). Catalase (CAT), superoxide dismutase (SOD), and peroxidases (POX) are among the enzymatic antioxidants that regulate ROS metabolism (Gill & Tuteja, 2010). Nonenzymatic components include ascorbic acid (AsA), tocopherol, flavonoids, glutathione, carotenoids, lipids, and phenolic compounds, which mitigate oxidative damage by scavenging free radicals or by working together with the enzymatic players to achieve antioxidant activity via the utilization of H_2_O_2_ (Nadarajah, 2020). Elevated H_2_O_2_ levels are associated with resistance of transgenic potato to *Erwinia carotovora* and *Phytophthora infestans* (Kuźniak & Urbanek, 2000), thiamine IR in rice against *M*. *graminicola* (Huang et al., 2016), ozonated water IR in tomato against *Meloidogyne incognita* (Veronico et al., 2017), and piperonylic acid IR against pest and diseases in tomato (Desmedt et al., 2021).

Next to ROS and antioxidant signalling, phytohormones are another group of regulators of plant responses to biotic and abiotic stresses (Denancé et al., 2013). Salicylic acid (SA), jasmonic acid (JA), and ethylene (ET) form the central backbone of plant immunity (De Vleesschauwer et al., 2013, 2014; Spoel & Dong, 2008). SA regulates various aspects of plant growth and development in addition to its role as activator of defence genes (Klessig et al., 2018). Establishment of systemic acquired resistance (SAR)—a specific type of IR triggered by necrotizing pathogens that leads to resistance in systemic tissues (De Kesel et al., 2021)—involves the generation and transport of signals via the phloem to distal tissues (Guedes et al., 1980), among which SA is a central component of SAR (Gao et al., 2015). IR activated by plant growth‐promoting rhizobacteria, necrotizing pathogens, and several chemical compounds, including β‐aminobutyric acid, ascorbate oxidase, and piperonylic acid, involves SA accumulation in treated plants (Desmedt et al., 2021; Jakab et al., 2001; Klessig et al., 2018; Singh et al., 2020b).

The antioxidant AsA regulates various plant physiological processes (Hossain et al., 2018). AsA reacts with ROS generated during stress to form monodehydroascorbate (MDHA), which then dissociates to dehydroascorbate (DHA). MDHA and DHA again get reduced to AsA by monodehydroascorbate reductase and DHA reductase, respectively. This AsA–DHA cycle is highly important for plant growth and development (Suekawa et al., 2018), as well as (a)biotic stress tolerance (Boubakri, 2018; Veljović‐Jovanović et al., 2018). DHA performs unique functions like cell cycle progression sensing and regulation, and modulation of metal stress responses, and DHA adducts seem to be involved in oxidative stress‐mediated cellular toxicity (Miret & Müller, 2018; Potters et al., 2000). The changes in the pool and ratio of the AsA/DHA redox pair by both growth and environmental cues modulate gene expression and protein levels, resulting in increased stress tolerance (Miret & Müller, 2018).

Previously, we showed that oxidation of AsA by exogenous application of ascorbate oxidase activates systemic defence mechanisms against plant‐parasitic nematodes in rice and sugar beet (Singh et al., 2020b, 2020a). This led to the hypothesis that the oxidized product of AsA, that is, DHA, might stimulate IR in plants. In the current research paper, we have confirmed this hypothesis and investigated the mechanisms of DHA‐based induced resistance in rice against root‐knot nematode *M. graminicola* in detail by performing infection assays, transcriptional analyses, biochemical assays, and hormone measurements.

## 2 RESULTS

### 2.1 DHA reduces plant susceptibility in rice against *M. graminicola*

**FIGURE 1:**
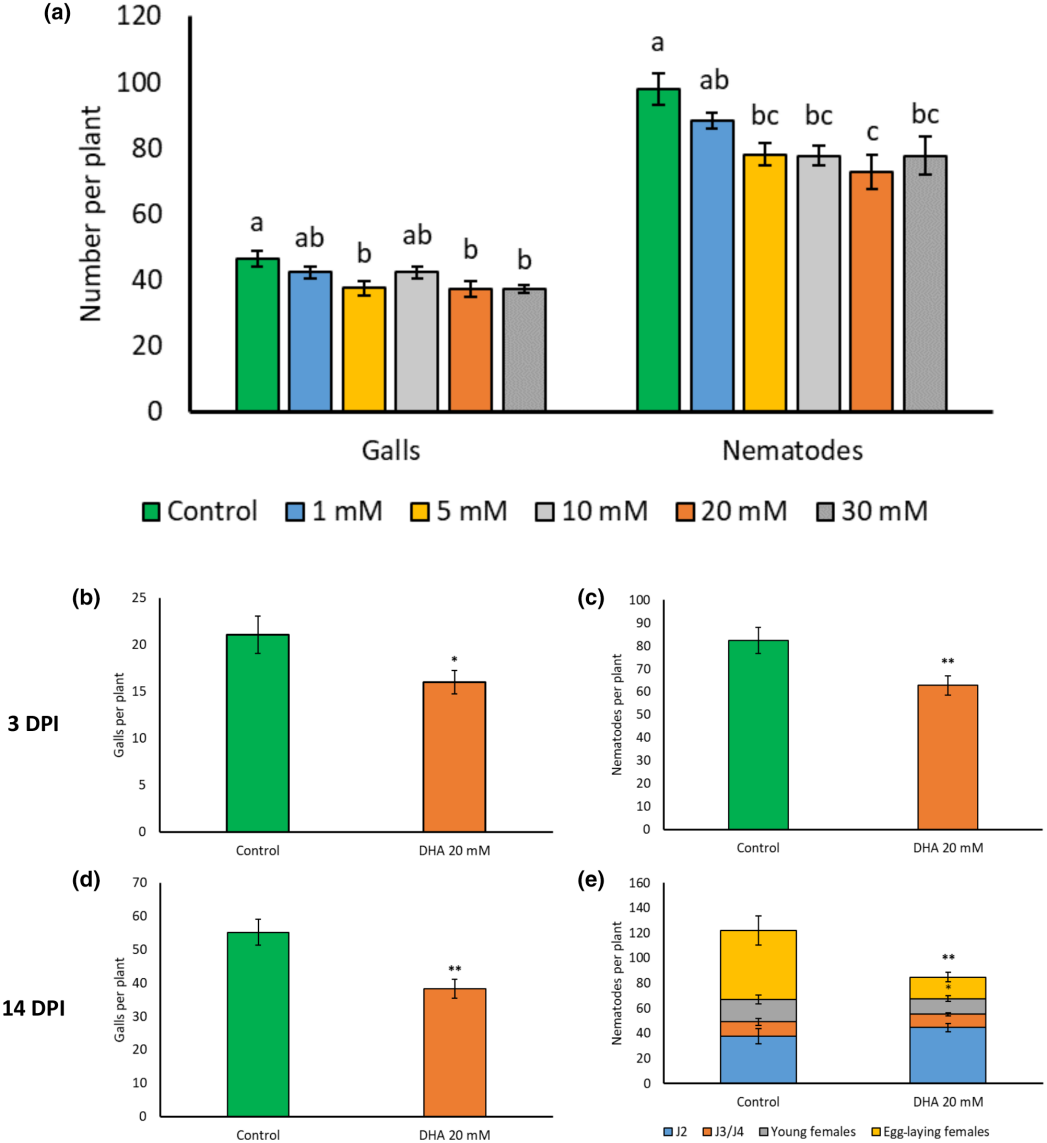
Effect of foliar dehydroascorbate (DHA) treatment on rice susceptibility to *Meloidogyne graminicola*. Two‐week‐old rice plants were treated with DHA followed by nematode inoculation (250/plant) 1 day posttreatment (DPT). (a) Effect of 1, 5, 10, 20, or 30 mM DHA on plant susceptibility to *M. graminicola* recorded 14 days after nematode inoculation (14 DPI). Effect of 20 mM DHA on (b) galls and (c) nematodes recorded 3 DPI. Effect of 20 mM DHA on (d) galls and (e) nematodes recorded 14 DPI. Bars represent the means and standard error of eight replicates. The whole experiment was independently repeated three times, providing confirmatory results. Different letters on error bars within a group indicate a statistically significant difference (Duncan's multiple range test, α = 0.05). Asterisks on error bars indicate statistically significant differences with the mock‐treated control plants (Student's *t* test, *p < 0.05, **p < 0.01).

In an experiment for the evaluation of different concentrations of DHA, we observed that foliar application of 5–30 mM DHA was effective in reducing susceptibility of rice roots to *M. graminicola*, with 20 mM being most effective (Figure 1a,b). The number of second‐stage juveniles (J2) was significantly lower in DHA‐treated plants versus mock‐treated control plants at 3 days postinoculation (DPI), indicating that nematode penetration is hampered (Figure 1c). Furthermore, numbers of galls, nematodes, and egg‐laying females were significantly lower in DHA‐treated plants versus mock‐treated control plants at 2 weeks postinoculation, revealing that nematode development is also affected (Figure 1d,e). These data confirm and extend the previous observations reported in Singh et al. (2020a).

Foliar treatment with DHA did not cause negative effects on rice growth up to a concentration of 30 mM (Figure S1). To confirm this observation and investigate potential long‐term effects, a greenhouse experiment with biweekly 20 mM DHA treatments was executed on two rice cultivars, but again no negative effects were observed (Figure S2). However, a slight positive effect on plant growth and yield was observed in DHA‐treated plants (Figure S2).

### 2.2 DHA acts as stimulant of systemic induced resistance in rice

To explore underlying molecular mechanisms of DHA‐induced systemic resistance, a transcriptome analysis was done on root tissues of rice plants treated with DHA at two concentrations (5 and 20 mM) and sampled at 1 and 4 days posttreatment (DPT), to be compared with same‐aged mock‐treated plants to identify differentially expressed genes (DEGs).

**FIGURE 2:**
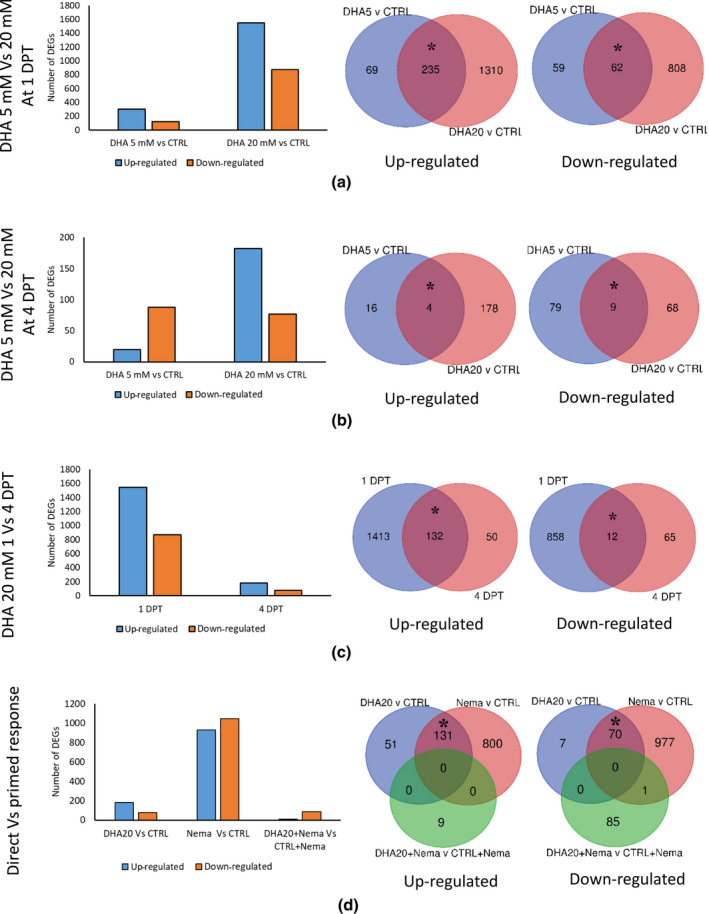
Differentially expressed genes (DEGs) in rice roots after foliar dehydroascorbate (DHA) treatment. Bar chart and Venn diagrams represent the number and overlap between DEGs after DHA treatment at 5 mM (DHA 5 mM vs. control) and 20 mM (DHA 20 mM vs. control) at (a) 1 and (b) 4 days posttreatment (DPT). (c) Number of DEGs after 20 mM DHA treatment (DHA 20 mM vs. control) at 1 and 4 DPT. (d) Number of DEGs after DHA treatment (DHA 20 mM vs. control) and in the nematode‐inoculated vs. control and 20 mM DHA + nematode vs. mock‐treated + nematode groups at 4 DPT/3 days postinoculation. Asterisks in the Venn diagram indicate statistically significant overlap between two gene lists (χ^2^ test, *p < 0.01).

A more robust transcriptional response was observed at the higher (20 mM) concentration (Figure 2a,b) than at 5 mM. A total of 425 and 108 DEGs were observed at 1 and 4 DPT with 5 mM DHA, while 2415 and 259 DEGs were detected at those time points in plants treated with 20 mM DHA (Figure 2a,b). Many DEGs were common between 5 and 20 mM DHA‐treated plants (χ^2^ = 3052.6, *p* = 2.2 × 10^−16^; χ^2^ = 927.98, *p* = 2.2 × 10^−16^ for up‐ and down‐regulated sets, respectively), while additional DEGs were only detected at the higher concentration (20 mM; Figure 2a,b). Most shared DEGs among 5 and 20 mM treatment groups were related to basic biological processes (Table S3a), while many stress/defence‐related GO terms were additionally enriched on treatment with the 20 mM DHA concentration (Table S3c). A more robust transcriptional response was observed at the earliest time point (1 DPT: 5 mM, 425 DEGs; 20 mM, 2415 DEGs) in comparison with the later time point (4 DPT: 5 mM, 108 DEGs; 20 mM, 259 DEGs) regardless of the concentration (Figure 2a–c). Several processes in the plant's basic metabolism, stress responses, immunity, antioxidant activity, and secondary metabolism were enriched on DHA treatment (Tables [Link], [Link], [Link]). DHA treatment led to increased expression of genes involved in JA, SA, ET, and other hormone pathways (Tables 1 and S3) as well as the phenylpropanoid and (di)terpenoid pathways (Table S5). Together, these data indicate that DHA induces transcriptional changes consistent with induced systemic resistance in rice.

**TABLE 1:** Overview of the root expression pattern of salicylic acid (SA) biosynthesis and responsive/signalling genes, based on mRNA‐Seq data of dehydroascorbate (DHA) 20 mM plants analysed 1 and 4 days after treatment and nematode‐inoculated plants in comparison with control plants

Gene name	Gene ID	DHA 20 mM vs. control (1 DPT)	DHA 20 mM vs. control (4 DPT)	Nematode vs. control (4 DPT/3 DPI)
SA biosynthesis				
OsPAL1	Os02g0626100	0.30	0.12	0.14
OsPAL2	Os02g0626400	**0.80**	−0.22	−0.31
OsPAL3	Os02g0626600	**1.64**	0.31	0.35
OsPAL4	Os02g0627100	**2.19**	0.23	0.18
OsPAL5	Os04g0518100	−0.03	−0.13	−0.12
OsPAL6	Os04g0518400	0.15	−0.01	−0.39
OsPAL7	Os05g0427400	**1.38**	0.27	**1.01**
OsPAL9	Os12g0520200	0.90	0.38	0.11
OsCM	Os01g0764400	**1.03**	0.18	0.35
OsICS1	Os09g0361500	**−1.02**	−0.29	**−0.70**
OsAIM1	Os02g0274100	0.25	0.21	0.33
CBP‐like family protein	Os12g0556200	**1.17**	0.04	−0.43
OsMESL	Os07g0603600	**0.64**	−0.12	−0.17
α/β‐hydrolase family protein	Os05g0370700	**2.48**	−0.19	−0.17
Similar to SA‐binding protein 2	Os01g0787600	**1.97**	0.51	0.84
SA‐responsive/signalling				
OsPR1a	Os07g0129200	**1.81**	**1.99**	**1.30**
OsPR1‐73	Os07g0127600	**4.55**	**2.91**	**1.01**
OsPR1b	Os07g0127700	**3.42**	**1.16**	0.96
OsPR3	Os03g0667100	0.48	−0.49	**−0.88**
OsPR5	Os03g0663500	**2.95**	**1.11**	2.05
OsPR10	Os12g0555000	**4.44**	**0.96**	**1.18**
OsPR10a	Os12g0555500	**3.86**	**0.64**	**1.09**
OsWRKY13	Os01g0750100	−0.01	−0.29	**−0.51**
OsWRKY45	Os05g0322900	0.43	−0.22	**−0.61**
OsWRKY62	Os09g0417800	0.35	−0.47	**−1.08**
OsWRKY67	Os05g0183100	**1.46**	0.26	0.12
OsWRKY76	Os09g0417600	0.04	−0.31	**−1.21**
OsNLA1	Os07g0673200	**0.92**	−0.24	**−0.57**
OsAOX1A	Os04g0600200	**0.94**	0.19	0.29
OsAOC	Os03g0438100	**0.87**	−0.32	**−0.42**
OsLTPd4	Os07g0290200	**0.64**	−0.32	−0.24
Similar to blight‐associated protein p12	Os09g0472900	**1.82**	**1.50**	**0.71**
OsPIOX	Os12g0448900	**1.19**	0.28	**1.05**
Similar to MAC	Os02g0475300	**0.72**	0.06	−0.07
Similar to NAC domain protein	Os01g0816100	**2.14**	−0.01	0.32
TIFY11D domain‐containing protein	Os10g0392400	**1.76**	−0.09	0.31

*Notes*: The table shows the log_2_ fold change (FC) of the gene expression in treated roots versus control. Values indicated in bold represent significant induction (FDR < 0.05). Genes were selected based on GO association with the SA pathway. Control, mock‐treated control plants.

Abbreviations: AIM, abnormal inflorescence meristem; AOC, allene oxide cyclase; AOX, alternative oxidase; CBP, calmodulin binding protein; CM, chorismate mutase; DPI, days postinoculation; DPT, days posttreatment; ICS, isochorismate synthase; LTPd4, nonspecific lipid transfer protein d4; MAC, membrane attack complex component; MESL, methyl esterase‐like; NAC, no apical meristem (NAM), ATAF1–2, and cup‐shaped cotyledon (CUC); NLA, nitrogen limitation adaptation; PAL, phenylalanine ammonia‐lyase; PR, pathogenesis‐related; PIOX, pathogen‐inducible oxygenase.

A large number of DEGs (1978) were detected in response to nematode infection. The DEGs were enriched for stress‐related GO terms (Figure 2d and Tables [Link], [Link], [Link]), confirming previous observations of transcriptional reprogramming by *M. graminicola* infection in rice (Kyndt et al., 2012a). Many DEGs were shared between DHA‐treated and nematode‐infected groups (χ^2^ = 2832.3, *p* = 2.2 × 10^−16^; χ^2^ = 1710, *p* = 2.2 × 10^−16^ for up‐ and down‐regulated sets, respectively; Figure 2d), indicating similarities in the plant responses to both stimuli. This might be because nematode infection has been shown to cause DHA accumulation (Singh et al., 2020a). These shared DEGs were enriched in GO terms associated with, for example, abscisic acid (ABA) binding, hormone binding, chitinase activity, oxidoreductase activity, defence response, and response to stress (Tables S2 and S3). Importantly, the nematodes, which are well known to interfere with plant metabolism and defence (Ji et al., 2013; Kyndt et al., 2012a), seemed less able to do so when plants were pretreated with DHA (Figures S3 and S4, and Tables S2 and S5), in line with decreased rice susceptibility on DHA treatment (Figure 1). A stronger transcriptional response was observed in response to DHA treatment (259 DEGs) when compared with DHA‐treated + nematode‐inoculated plants (95 DEGs) (Figure 2d). This indicates that the resistance induced by DHA (DHA‐IR) is primarily based on direct activation of plant immunity rather than on defence priming.

### 2.3 DHA‐induced resistance involves activation of ROS metabolism

**FIGURE 3:**
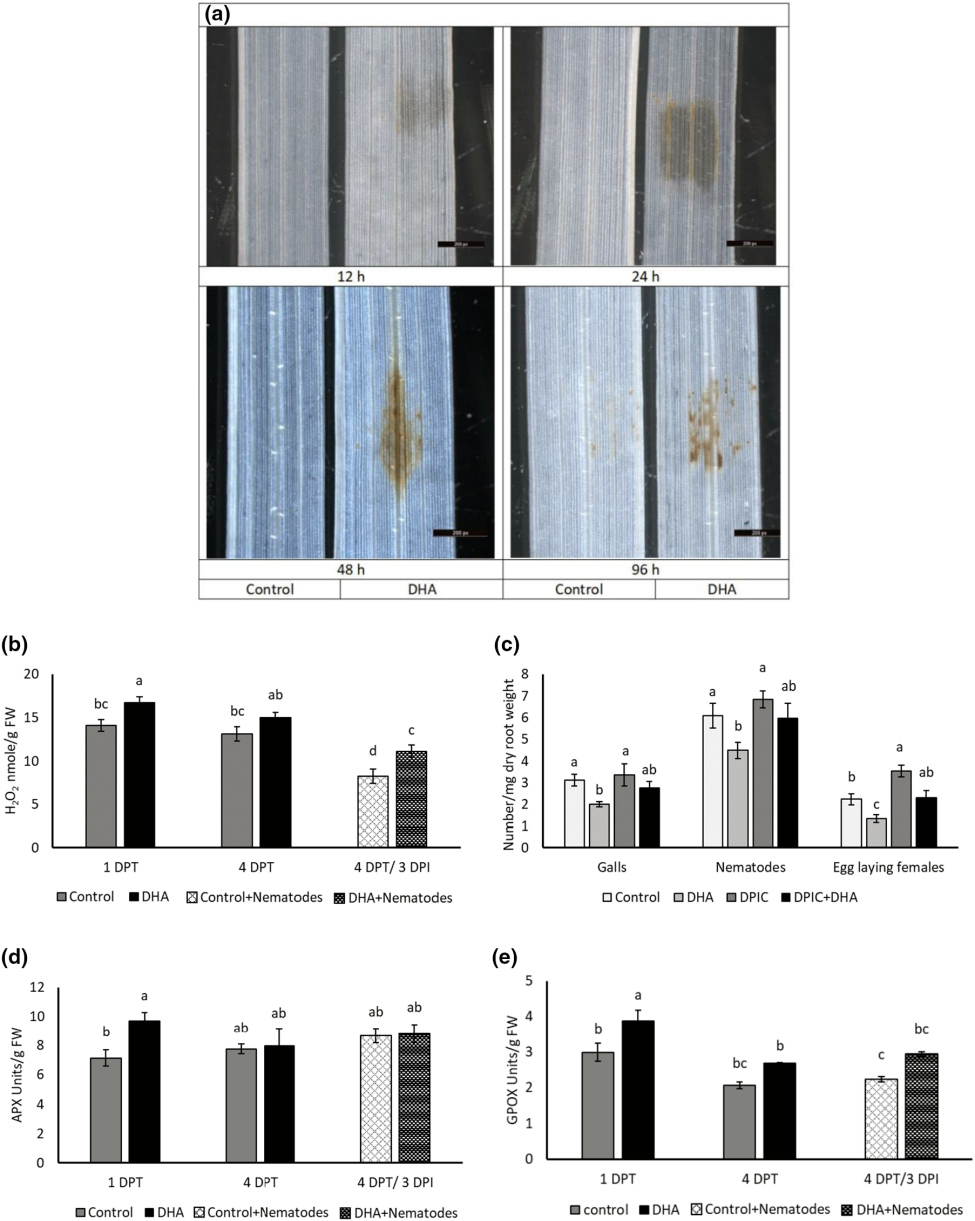
Effect of 20 mM dehydroascorbate (DHA) treatment on reactive oxygen species (ROS) metabolism in rice. (a) Qualitative detection of H_2_O_2_ in 20 mM DHA‐treated plants. Brown spots following 3,3′‐diaminobenzidine staining indicate the presence of H_2_O_2_ in rice leaves at 12, 24, 48, and 96 h of DHA or mock treatment. (b) H_2_O_2_ content in root tissues of 20 mM DHA‐treated plants at 1 and 4 days posttreatment (DPT) and at 3 days after nematode inoculation (DPI) in DHA‐treated plants (4 DPT/3 DPI). Error bars indicate the *SE* of six biological replicates, each containing a pool of four or five plants. (c) Nematode infection experiment using the ROS inhibitor diphenyleneiodonium chloride (DPIC) alone or in combination with 20 mM DHA. At 1 DPT, 250 second‐stage juveniles of *Meloidogyne graminicola* were inoculated per plant. Galls, nematodes, and egg‐laying females were counted at 14 DPI. Error bars indicate the *SE* of eight replications. The whole experiment was independently repeated twice, providing confirmatory results. (d) Ascorbate peroxidase (APX) and (e) guaiacol peroxidase (GPOX) activity in root tissues of 20 mM DHA‐treated rice plants at 1 and 4 DPT and 4 DPT/3 DPI. Error bars indicate the *SE* of six biological replicates, each containing a pool of four or five plants. Different letters indicate a statistically significant difference (Duncan's multiple range test, α = 0.05).

mRNA sequencing (mRNA‐Seq) data indicated significant GO enrichment in antioxidant and peroxidase activity on DHA treatment (Tables [Link], [Link], [Link]). The local accumulation of H_2_O_2_ in rice plants on foliar DHA treatment was confirmed by 3,3′‐diaminobenzidine (DAB) staining at the site of application, where a dark‐brown polymerization product was observed in DHA‐treated leaf samples at all investigated time points (12–96 h; Figure 3a). This shows that H_2_O_2_ was generated in leaves early on DHA treatment, and its levels remained high for at least 4 days after DHA treatment.

To quantitatively assess whether H_2_O_2_ accumulation on foliar DHA treatment has systemic effects, H_2_O_2_ content was determined in root tissues using the FOX assay. A significantly higher level of H_2_O_2_ was observed at 1 DPT with 20 mM DHA compared to mock‐treated plants (Figure 3b). H_2_O_2_ was also significantly increased at 3 DPI in DHA‐treated nematode‐inoculated plants in comparison with mock‐treated nematode‐inoculated plants. This confirms increased root H_2_O_2_ levels in DHA‐treated plants under both uninfected and nematode‐infected conditions.

To confirm the role of ROS accumulation in DHA‐IR, an infection experiment was conducted on plants treated with ROS inhibitors alone and in combination with DHA. Previous reports showed that plant treatment with DPIC leads to reduced ROS production (Li & Trush, 1998) and increased susceptibility to RKNs (Singh et al., 2020a). Treatment of plants with DHA led to reduced plant susceptibility to *M*. *graminicola* (Figure 3c). However, when DHA was applied in combination with DPIC, DHA could no longer induce resistance against *M. graminicola* (Figure 3c). Similar results were obtained using other ROS inhibitors, namely catalase and DMTU, where combined application of these inhibitors with DHA impaired DHA‐IR (Figure S5).

The role of peroxidases in DHA‐IR was studied by measuring ascorbate peroxidase (APX) and guaiacol peroxidase (GPOX) activity on DHA treatment. Induction of APX (Figure 3d) and GPOX (Figure 3e) activity was observed in root tissues of DHA‐treated plants at 1 DPT. GPOX activity was also significantly induced at 4 DPT as well as in in DHA‐treated nematode‐inoculated plants in comparison with mock‐treated nematode‐inoculated plants (Figure 3e). Taken together, our data reveal that in planta production of H_2_O_2_ and its metabolism is one of the major biochemical mechanisms underlying DHA‐IR.

### 2.4 DHA activates induced resistance in rice through activation of the SA pathway

**FIGURE 4:**
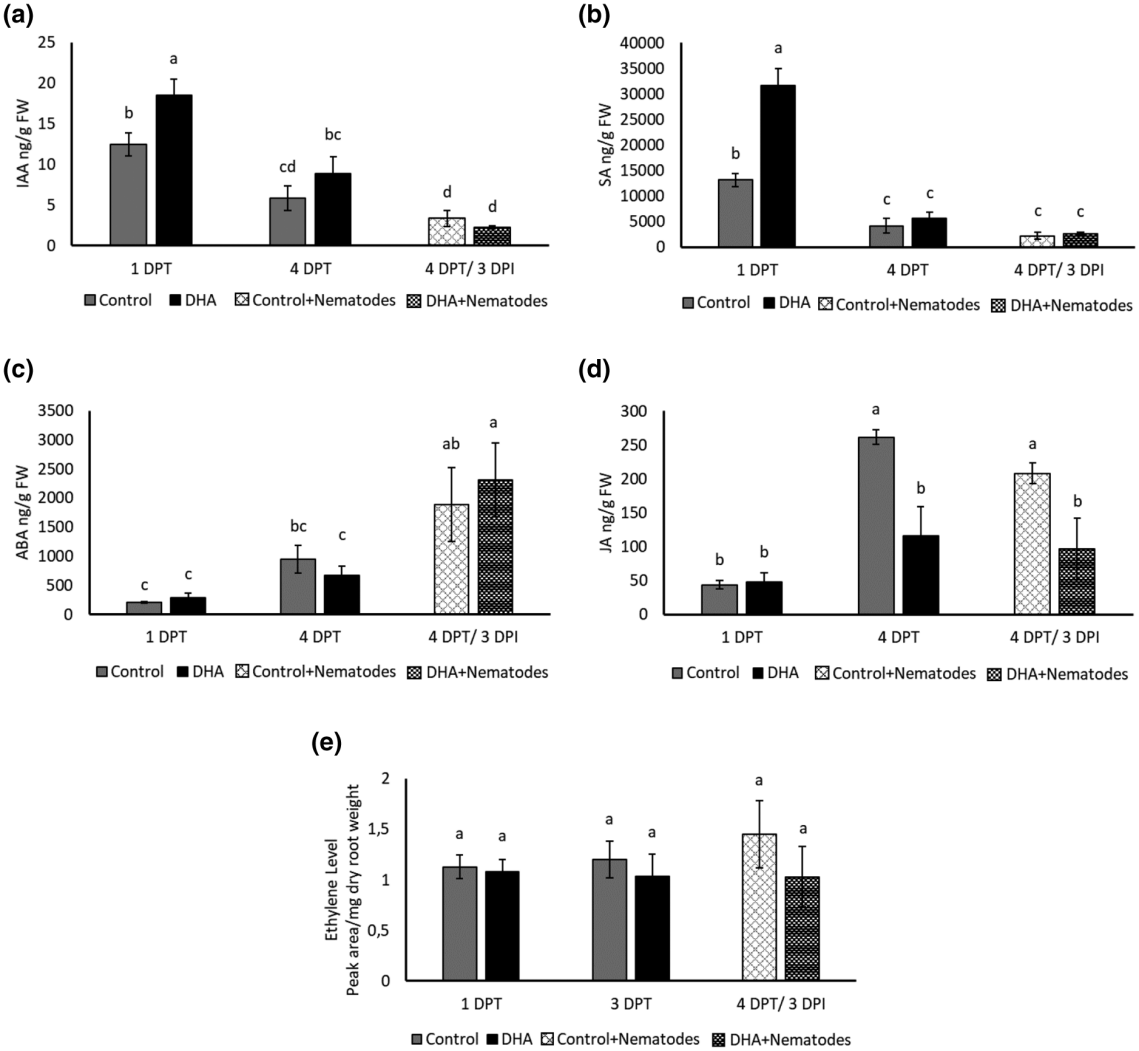
Hormone levels in roots of rice plants treated with 20 mM dehydroascorbate (DHA) vs. mock‐treated control plants, measured at 1 and 4 days posttreatment (DPT) and 3 days after nematode inoculation in DHA‐treated plants (4 DPT/3 DPI). (a) Indole‐3‐acetic acid (IAA), (b) salicylic acid (SA), (c) abscisic acid (ABA), (d) jasmonic acid (JA), and (e) ethylene. Error bars indicate the *SE* of six biological replicates, each containing a pool of four or five plants. Different letters on error bars indicate a statistically significant difference (Duncan's multiple range test, α = 0.05).

mRNA‐Seq data of DHA‐treated plants indicated the disturbance of phytohormone‐related genes (Figure S3, and Tables 1 and S5). Hence, the levels of indole‐3‐acetic acid (IAA), SA, ABA, JA, and ET were measured in roots of DHA‐treated plants. A significantly higher concentration of IAA and SA was observed on DHA treatment at 1 DPT (Figure 4a,b), while a significantly lower quantity of JA was observed at 4 DPT as well as at 3 DPI in DHA‐treated plants (Figure 4d). No significant differences were observed in levels of ABA and ET between DHA‐treated plants and mock‐treated plants (Figure 4c,e).

**FIGURE 5:**
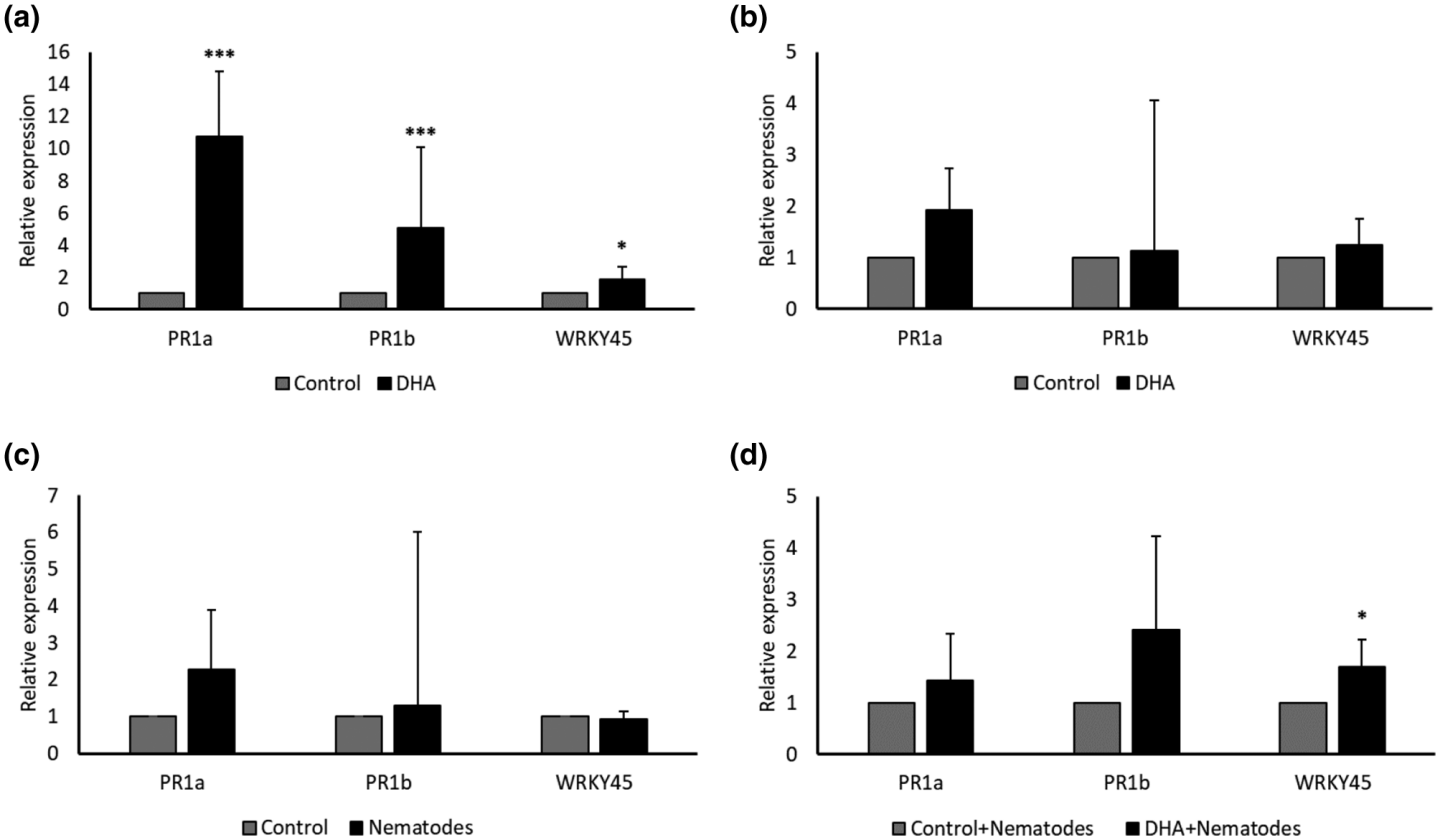
Relative expression of salicylic acid (SA) marker genes *PR1a*, *PR1b*, and *WRKY45* in root tissues of rice plants treated with dehydroascorbate (DHA) or infected with *Meloidogyne graminicola*, analysed at (a) 1 and (b) 4 days after DHA treatment (DPT) and (c) 3 days after nematode inoculation (DPI) in mock‐treated and (d) 3 DPI in DHA‐treated plants. Expression levels were determined by reverse transcription‐quantitative PCR using three technical and three biological replicates, and are expressed relative to mock‐treated plants. Error bars indicate the 95% confidence interval. Gene expression levels were normalized using two internal reference genes and statistical analysis was done in REST 2009. Asterisks indicate significant differential expression in comparison with mock‐treated plants (*p < 0.05, ***p < 0.001).

**FIGURE 6:**
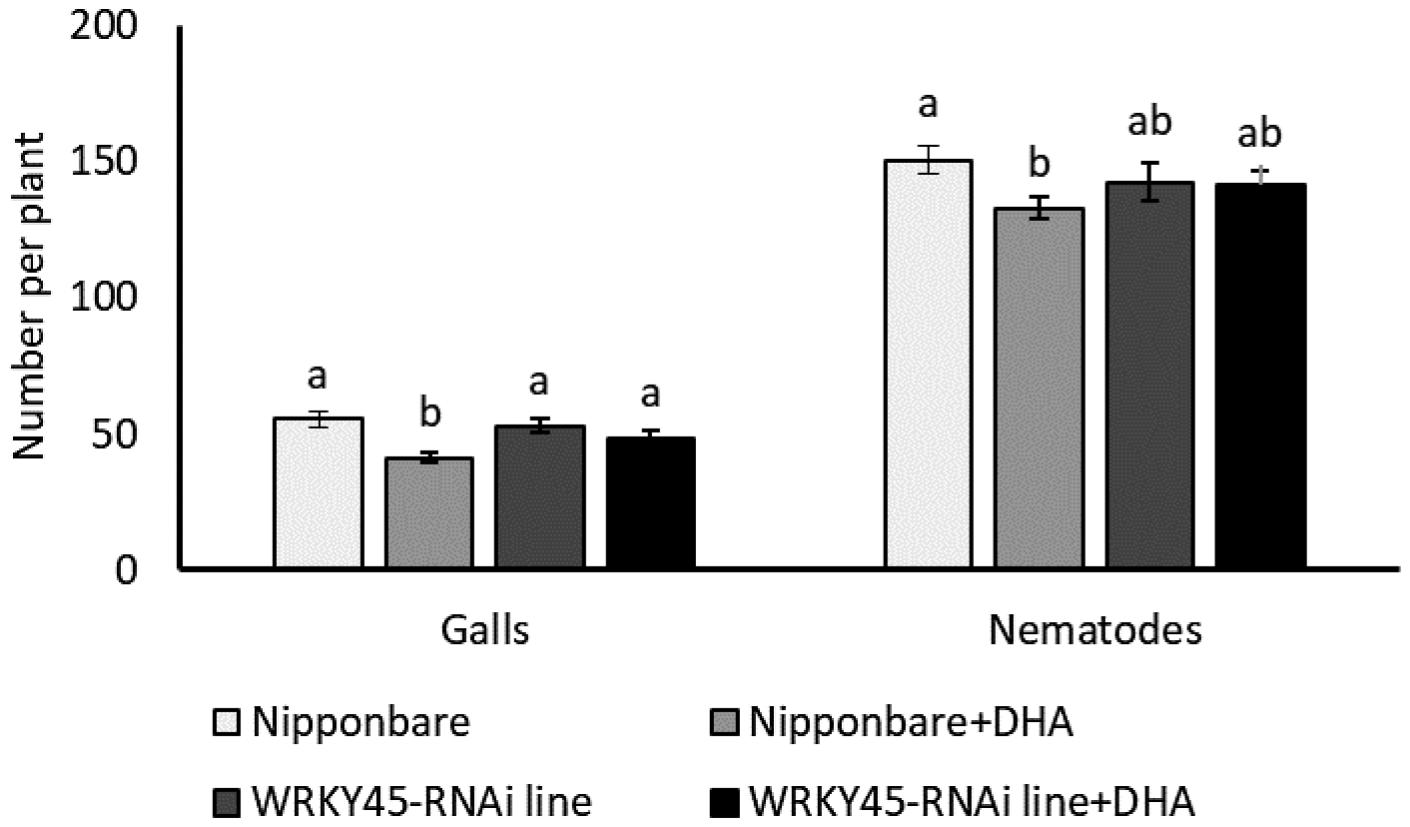
Effect of dehydroascorbate (DHA) on rice susceptibility to *Meloidogyne graminicola* in *WRYK45*‐RNAi line and wild‐type Nipponbare. Two‐week‐old rice plants were treated with 20 mM DHA followed by 250 J2 nematode inoculation at 1 day posttreatment. Galls and nematodes were recorded 14 days postinoculation. Error bars indicate the *SE* from 12 replicates. The whole experiment was independently repeated twice, providing confirmatory results. Different letters indicate a statistically significant difference (Duncan's multiple range test, α = 0.05).

Activation of the SA pathway was further validated by reverse transcription‐quantitative PCR (RT‐qPCR)‐based expression analysis of SA marker genes: PR (pathogenesis‐related) genes *PR1a* and *PR1b*, and transcription factor *WRKY45*. A significantly higher expression of *PR1a*, *PR1b*, and *WRKY45* was observed at 1 DPT in roots of DHA‐treated plants compared to mock‐treated plants (Figure 5a), in line with mRNA‐Seq data (Table 1) and SA measurements (Figure 4b). No significant differences in the expression of these genes were detected on nematode infection (Figure 5c). However, a significantly higher *WRKY45* expression was observed at 3 DPI in DHA‐treated plants in comparison with mock‐treated nematode‐inoculated plants, indicating a primed activation of this gene (Figure 5c,d). To further investigate the importance of *WRKY45* in DHA‐IR, a nematode infection experiment was conducted using SA signalling‐deficient *WRKY45*‐RNAi line (Shimono et al., 2007). The *WRKY45*‐RNAi line was not different in nematode susceptibility (Figure 6), as previously reported (Ji et al., 2015). Treatment of wild‐type Nipponbare plants with DHA led to reduced plant susceptibility to *M. graminicola* (Figure 6), whereas DHA treatment in *WRKY45‐*RNAi plants impaired DHA‐IR (Figure 6). These results indicate that DHA‐IR is dependent on SA signalling through *WRKY45*.

## 3 DISCUSSION

AsA is one of the most abundant water‐soluble antioxidants in plants and acts as a key regulator in growth and development (Hossain et al., 2018), and abiotic (Billah et al., 2017; Farooq et al., 2013; Kobayakawa & Imai, 2017; Wang et al., 2017; Xu & Huang, 2018) as well as biotic stress tolerance (Boubakri, 2018; Egan et al., 2007; Fujiwara et al., 2013; Li et al., 2016). Oxidation of AsA is an important event in the stress‐induced AsA:DHA cycle, catalysed by the enzymes ascorbate oxidase (AO) and APX (Green & Fry, 2005; Stevens et al., 2018). Recent evidence shows that oxidation of AsA by exogenous AO treatment activates systemic resistance against plant‐parasitic nematodes in rice and sugar beet (Singh et al., 2020b, 2020a). Here, we showed that the exogenous foliar application of DHA, the reversibly oxidized form of AsA, activates induced resistance in rice against *M. graminicola* and we investigated the mechanisms underlying DHA‐IR.

An early and rapid transcriptional response is one of the key features of IR (Chen et al., 2020; De Kesel et al., 2021; Desmedt et al., 2021). In accordance, a stronger transcriptional and (bio)chemical response was observed at 1 DPT versus 4 DPT (Figures 2, 3, 4) on DHA treatment. mRNA‐Seq revealed that DHA induced systemic transcriptional reprogramming in oxidative stress, phenylpropanoid, and defence hormone pathways (Tables 1 and S5). Similarly, piperonylic acid‐IR in tomato and chito‐oligosaccharide + oligogalacturonide‐IR in rice causes transcriptional reprogramming of the phenylpropanoid pathway, ROS metabolism, and SA signalling (Desmedt et al., 2021; Singh et al., 2019).

A significant reduction in nematode penetration was observed in roots of DHA‐treated rice plants (Figure 1c). This is indicative of prepenetration resistance, a situation in which nematodes are unable to enter the host plant due to, for example, absence of the metabolites needed for host recognition, repellent host exudates, or a physical barrier (Desmedt et al., 2020; Lee et al., 2017). Nematodes that fail to establish feeding sites either die or leave the hostile roots, as observed in β‐aminobutyric acid‐treated rice plants (Ji et al., 2015), and in RKN‐resistant cultivars in soybean (Herman et al., 1991) and alfalfa (Griffin & Elgin, 1977; Reynolds et al., 1970). In addition to hampered root penetration, DHA also affected nematode development (Figure 1e). Similarly, *Trichoderma*‐IR in tomato against *M. incognita* impairs both penetration and development, and this effect was found to be related to time‐dependent activation of both the SA and JA pathways (Martínez‐Medina et al., 2017).

Disturbance of phytohormone pathways was also observed on DHA treatment (Figures 4 and S4, and Tables 1 and S3), with SA and IAA accumulation at early time points, while JA depleted at 4 DPT. Knowing that auxins such as IAA promote plant susceptibility to parasitic nematodes (Goverse et al., 2000; Grunewald et al., 2009a, 2009b; Kyndt et al., 2016), while SA and JA have a time‐dependent defensive role against RKNs (Martínez‐Medina et al., 2017), these data point to a major role for SA in DHA‐IR against *M. graminicola* in rice (Figure 4b). Rice leaves contain very high basal levels of SA (Raskin et al., 1990) but studies have shown that this pathway can still be activated to achieve benzothiadiazole‐IR against *Magnaporthe oryzae* (Shimono et al., 2007). A significant induction of SA marker genes on DHA treatment (Table 1, Figure 5) supports the hormone measurements. The SA pathway is predominantly effective against biotrophic pathogens (Pieterse et al., 2009), including parasitic nematodes (Branch et al., 2004; Kammerhofer et al., 2015; Yang et al., 2018). Rice *WRKY45* plays a crucial role in benzothiadiazole‐induced and SA‐mediated defence signalling in blast resistance (Shimono et al., 2007). A significant induction of *WRKY45*expression was seen on DHA treatment 1 DPT and on RKN infection in DHA‐treated plants (Figure 5a,d). The impairment of DHA‐IR in the *WRKY45*‐RNAi line further supports the involvement of *WRKY45* in DHA‐based IR. Previously, we showed that foliar AO treatment leads to ET and JA accumulation in rice roots (Singh et al., 2020a), and SA accumulation in sugar beet (Singh et al., 2020b). Contrary to reports on SA–JA antagonism in *Arabidopsis* defence against aboveground pathogens (Pieterse et al., 2009), these hormones rather seem to collaborate in root defence against RKNs (Martínez‐Medina et al., 2017; Nahar et al., 2011). Here, activation of the JA pathway was only observed in the mRNA‐Seq dataset (Figure S3 and Table S5b), while lower endogenous levels were observed at 4 DPT (Figure 4d). These data are indicative of a negative feedback loop and suggest that the JA peak was probably already occurring before 1 DPT.

The defence responses underlying IR can be activated directly and/or be primed for augmented expression on stress exposure (De Kesel et al., 2021; Van Hulten et al., 2006). The IR‐stimulating chemical compound diproline leads to a direct activation of defence genes in rice (De Kesel et al., 2020), while AO (Singh et al., 2020a) and piperonylic acid (Desmedt et al., 2021) activate both direct and primed defence responses in rice and tomato, respectively. In our study, we observed a strong transcriptional response, as well as H_2_O_2_, APX, GPOX, and SA accumulation already at 1 DPT with DHA while only a modest response was detected on additional challenge of DHA‐treated plants with RKNs (Figure 2). These data suggest that DHA primarily directly activates plant immune pathways rather than priming their activation. Interestingly, despite the strong activation of plant immunity by DHA treatment, no growth defects were detected in regularly treated plants. This could be related to the higher IAA content on DHA treatment (Figure 4a), which could explain the slight positive effect on plant growth and yield (Figure S3).

The increased H_2_O_2_ production on DHA treatment (Figure 3a,b) and reduced efficacy of DHA‐IR in plants treated with ROS inhibitors (Figures 3c and S5) confirms that ROS contribute to DHA‐based IR. While both AsA and DHA are well‐known antioxidants (Dewhirst & Fry, 2018), the pro‐oxidant nature of both compounds has also been described (Kärkönen & Fry, 2006; Smirnoff, 2018). DHA degradation in planta can produce a range of products that give rise to H_2_O_2_ (Deutsch, 2000; Parsons et al., 2011; Smirnoff, 2018), which could influence defence responses and cell wall polymer cross‐linking processes that depend on H_2_O_2_ and peroxidases (Smirnoff, 2018). Stimulation of APX and GPOX on DHA treatment confirms the activation of ROS metabolism in DHA‐treated plants (Figure 3c,d). Such increased peroxidase activity has also been reported during the activation of IR by plant growth‐promoting rhizobacteria and chemical compounds (Anand et al., 2007; Naz et al., 2021; Retig, 1974; Survila et al., 2016; Yanti, 2015).

The plants were treated with DHA throughout our experiments by foliar application and various analyses such as mRNA‐Seq, and enzyme and hormone measurements were done on roots. This indicates that the defence response induced by DHA is systemic in nature. Systemic signalling is a common phenomenon in plant stress responses and is one of the key features of IR (Vlot et al., 2020). The compound responsible for the systemic spread in DHA‐IR remains unclear. Exogenous application of DHA is known to affect cellular AsA/DHA homeostasis in rice, with significant increases in DHA levels in shoots but not in roots (Singh et al., 2020a), indicating that a compound other than DHA is transported to the roots. Accumulation of H_2_O_2_ and SA in roots of DHA‐treated plants (Figures 3a,b and 4b) suggests they might play a role in systemic DHA‐IR, as systemic signalling by SA (Ament et al., 2010; Dempsey & Klessig, 2012; Koo et al., 2007; Rowen et al., 2017; Shulaev et al., 1997) and H_2_O_2_ (Miller et al., 2009) has been documented. Different lines of evidence indicate that ROS and SA interact in mounting plant defence (Herrera‐Vásquez et al., 2015). SA can increase H_2_O_2_ levels in plant tissues (Dat et al., 1998; Rao et al., 1997), and conversely SA accumulation can be induced by increased H_2_O_2_ levels (Chamnongpol et al., 1998). ROS signals are thus involved both upstream and downstream of SA signalling (Herrera‐Vásquez et al., 2015). Typically, H_2_O_2_ is an early signalling molecule in plant stress responses (Černý et al., 2018). In light of our understanding of the ROS burst as a trigger for SA signalling (Chaouch et al., 2010; Herrera‐Vásquez et al., 2015; Mammarella et al., 2015; Maruta et al., 2012; Noshi et al., 2012; Wrzaczek et al., 2013), we hypothesize that DHA treatment in rice causes increased accumulation of ROS, after which ROS activates SA signalling by a primed induction of *WRKY45*. This then leads to reduced rice susceptibility to *M. graminicola*.

## 4 EXPERIMENTAL PROCEDURES

### 4.1 Plant material and growth conditions

Seeds of rice *Oryza sativa* ‘Nipponbare’ (GSOR‐100; USDA) and the SA signalling deficient *WRKY45*‐RNAi line (Shimono et al., 2007) were germinated in the dark for 4 days at 30°C. The *WRKY45*‐RNAi line was confirmed to have a significantly lower expression of *WRKY45* compared to wild‐type Nipponbare (Figure S6). Sprouted seeds were transferred to polyvinyl chloride (PVC) tubes (diameter 3 cm, length 18 cm) containing SAP substrate (sand mixed with Absorbent Polymer AquaPerla; DCM) (Reversat et al., 1999). They were further grown in a rice growth room at 26°C under 12 h/12 h light/dark regime (150 μmol·m^−2^·s^−1^) and relative humidity of 70%–75%. Plants were watered three times a week with 10 ml of Hoagland's solution (Hoagland & Arnon, 1950).

### 4.2 Nematode culture, inoculation, and evaluation of plant susceptibility

A pure culture of *M. graminicola* was originally obtained from the Philippines (kindly provided by Professor Dirk De Waele, KU Leuven) and maintained on barnyard grass (*Echinochloa crus‐galli*). Second‐stage juveniles (J2) were extracted from infected plants following a modified Baermann funnel method (Whitehead & Hemming, 1965). Two‐week‐old plants were inoculated with 250 J2s or mock‐inoculated with water at 1 DPT. Plant susceptibility was assessed at 3 DPI by counting number of J2s penetrated in roots and at 2 weeks after nematode inoculation by counting galls, total nematodes, and egg‐laying females using the acid fuchsin staining technique (Byrd et al., 1983). Galls and nematodes were counted using a binocular stereomicroscope (SMZ1500; Nikon). All infection experiments were repeated at least twice, each time using 8–12 plants per treatment.

### 4.3 Chemical treatments

The concentration of DHA (l‐dehydroascorbic acid; Sigma‐Aldrich) was optimized for efficacy against *M. graminicola* and lack of phytotoxicity by evaluating 1, 5, 10, 20, or 30 mM DHA. The aboveground parts of each plant were sprayed until run‐off with a 6.25 ml of DHA solution or distilled water, both containing 0.02% (vol/vol) of Tween 20 (Sigma‐Aldrich) for efficient spread and uptake of chemicals (Nahar et al., 2011). Plants were inoculated with nematodes 1 DPT. Among these concentrations, a range of 5–30 mM DHA was effective in reducing rice susceptibility without negatively affecting plant growth (Figures 1a and S1). No supplementary reduction in plant susceptibility was observed beyond the 20 mM concentration. One low (5 mM) and one high (20 mM) effective DHA concentration was used for mRNA‐Seq analysis. A concentration of 20 mM DHA was used for all further experiments.

To evaluate potential long‐term effects on rice growth and yield, a greenhouse experiment was conducted using two different rice cultivars (Kitaake and Nipponbare). Plants were treated with 20 mM DHA at biweekly intervals throughout their life cycle. Mock‐treated control plants were maintained under the same conditions. Each treatment contained nine plants. Plant growth was evaluated by measuring shoot length at biweekly intervals. The number of tillers, panicles, and total seed weight per plant was recorded at the time of harvest.

To investigate the role of ROS in DHA‐IR, plants were foliarly treated with the ROS inhibitors diphenyleneiodonium chloride (NADPH oxidase inhibitor) (DPIC; Sigma‐Aldrich) at 50 μM, catalase (H_2_O_2_ scavenger) (Cat; Sigma‐Aldrich) at 2 mg/50 ml or dimethyl thiourea (H_2_O_2_ and OH^−^ scavenger) (DMTU; Sigma‐Aldrich) at 5 mM, applied either individually or in combination with 20 mM DHA as described above.

### 4.4 mRNA‐Seq and data analysis

mRNA‐Seq was done on the whole root system of rice plants after foliar treatment with DHA. Four treatment groups were made: DHA‐treated plants, mock‐treated control plants, DHA‐treated + nematode‐inoculated plants, and mock‐treated + nematode‐inoculated plants. Two‐week‐old rice plants were treated with DHA at 5 mM, DHA at 20 mM or distilled water both containing 0.02% (vol/vol) Tween 20 as a foliar application. DHA was applied on the aboveground tissues and root samples were used for mRNA sequencing to evaluate the systemic response induced by foliar DHA treatment. Samples were collected at 1 and 4 DPT. To evaluate if DHA activates defence priming, one group of 20 mM DHA or mock‐treated plants was additionally exposed to biotic stress by inoculating 250 J2s of *M. graminicola* at 1 DPT and samples were collected 3 DPI (4 DPT/3 DPI and 3 DPI in case of mock treatment). For each treatment, three independent biological replicates, each containing the pooled root material of four plants, were used.

RNA was extracted using the RNeasy Plant Mini kit (Qiagen). RNA integrity was assessed using the Agilent 2100 Bioanalyzer System and approximately 1 μg was used for 3′ mRNA‐Seq library preparation using the QuantSeq 3′ mRNA‐Seq Library Prep Kit FWD (Lexogen). To minimize lane effects, samples were multiplexed using the Multiplexing Sample Preparation Oligo Kit (Illumina). Size selection was performed on a 2% agarose gel (low range ultra agarose; Bio‐Rad). The denatured library was diluted to a final concentration of 6 pM and loaded into a flow cell (Illumina). After cluster generation, the multiplexed library was sequenced on an Illumina NextSeq 500 System (75 cycles, single‐end, high output).

Reads were trimmed with Trimmomatic v. 0.36 (Bolger et al., 2014) and mapped against the *Oryza sativa* subsp. *japonica* ‘Nipponbare’ reference genome (build MSU7.0) using STAR v. 2.5.2a (Dobin et al., 2013). Only uniquely mapped reads were used for further analysis. BAM files of multiplexed samples were merged using samtools v. 1.3. Count tables were generated by the ‘Summarize Overlaps’ function in the Genomic Alignments R package v. 1.16.0 (Lawrence et al., 2013). The baseline characteristics of RNA‐Seq data are provided in Table S6. Differential gene expression analysis was performed using DESeq2 v. 1.20 (Love et al., 2014) with the annotations from the Rice Annotation Project Database v. 38. Genes with a false discovery rate (FDR) <0.05 were considered differentially expressed compared to the control group. The complete list of differentially expressed genes for all comparisons is provided in Table S7.

Gene Ontology (GO) enrichment analysis on DEGs was performed using g:Profiler v. e102_eg49_p15_7a9b4d6 with g:SCS multiple testing correction and a significance threshold of 0.05 (Raudvere et al., 2019). MapMan (Thimm et al., 2004) was used to visualize expression of genes involved in various metabolic pathways. The WSR test (with Benjamini–Hochberg correction) was used to test the statistical significance of differential expression of these pathways. The unprocessed mRNA‐Seq data can be retrieved from NCBI as BioProject PRJNA767540.

### 4.5 Biochemical assays

In each biochemical assay described below, root samples were collected from four treatment groups: 20 mM DHA‐treated plants, mock‐treated control plants, 20 mM DHA‐treated + nematode‐inoculated plants, and mock‐treated + nematode‐inoculated plants. Roots were snap‐frozen and finely ground in liquid nitrogen.

#### 4.5.1 Hydrogen peroxide concentration

Root H_2_O_2_ content was determined using the modified ferrous oxidation‐xylenol orange (FOX) assay (Awwad et al., 2019; El‐Shabrawi et al., 2010; Kaur et al., 2016). Tissue (100 mg) was homogenized in trichloroacetic acid solution (1 ml, 2.5% wt/vol) and centrifuged at 18,000 × *g* at 4°C for 9 min. The supernatant (“plant extract”) was used for quantitative measurement of H_2_O_2_. FOX reagent was prepared by mixing 100 volumes of reagent A (100 mM sorbitol + 125 μM xylenol orange in distilled water + 1% ethanol) with 1 volume of reagent B (25 mM ferrous ammonium sulphate [Mohr's salt] + 2.5 M H_2_SO_4_ in distilled water). Solutions were freshly prepared and used within 2 h. Plant extract (0.2 ml) was mixed with FOX reagent (1 ml), vortexed, and incubated for 30 min in the dark at room temperature. Absorbance was measured at 595 nm in three technical replicates. The amount of H_2_O_2_ was estimated using a standard curve with known concentrations of H_2_O_2_. We analysed six biological replicates, each containing the pooled material of at least four plants.

#### 4.5.2 Histochemical H_2_O_2_ detection using DAB assay

Histochemical detection of cellular H_2_O_2_ was performed using DAB as described by Daudi and O'Brien (2012). The second leaf of 2‐week‐old rice plants was treated by applying two 15‐μl droplets of 20 mM DHA or distilled water and sampled at 12, 24, 48, or 96 h after treatment. Leaves were cut into 1–2‐cm pieces and submerged into the DAB solution (1 mg/ml) in a six‐well plate using tweezers and scissors. The dipped samples were vacuum‐infiltrated for 5 min at 60 kPa and incubated at room temperature for 4 h in the dark on an orbital shaker. Leaves were cleared using ethanol, acetic acid, and glycerol in a 3:1:1 ratio at 95°C in a hot water bath for 15–20 min. The deep‐brown polymerization product of DAB and H_2_O_2_ was visualized using a stereomicroscope (SMZ1500; Nikon).

#### 4.5.3 APX activity

APX activity was measured by monitoring the decrease in absorbance at 290 nm as described by Nakano and Asada (1981), with some modifications described by Hong et al. (2018), Jiang et al. (2016), and Liu et al. (2019). Tissue (100 mg) from each sample was homogenized with 1 ml of 50 mM sodium phosphate buffer (pH 7.0) containing 0.1 mM EDTA, 5 mM β‐mercaptoethanol, 2% polyvinylpyrrolidone (PVP 40), 1 mM phenylmethanesulfonyl fluoride (PMSF), and 5 mM AsA. The homogenate was centrifuged at 18,000 × *g* at 4°C for 20 min, and the supernatant “plant extract” was used for estimating APX activity. The reaction mixture contained 50 mM sodium phosphate buffer (pH 7.0), 0.5 mM AsA, 2 mM H_2_O_2_, 0.1 mM EDTA, and 0.1 ml of plant extract in a total volume of 3 ml. The reaction was started by adding H_2_O_2_, and APX activity was determined by measuring the decrease in absorbance at 290 nm, assuming an absorption coefficient of 2.8 mM^−1^·cm^−1^, due to AsA oxidation over 3 min, in three technical replicates. One unit of APX activity was expressed as the amount of enzyme that can oxidize 1 mM of AsA per minute. We analysed six biological replicates, each containing the pooled material of at least four plants.

#### 4.5.4 GPOX activity

GPOX activity was measured by monitoring the increase in absorbance at 470 nm as described by Velikova et al. (2000) with minor modifications. One hundred milligrams of each sample was homogenized in 1 ml of 100 mM potassium phosphate buffer (pH 7.0) containing 0.1 mM EDTA, 2% PVP 40, and 1 mM PMSF. The homogenates were centrifuged at 18,000 × *g* at 4°C for 20 min, and the supernatant (plant extract) was used for estimating GPOX activity using a reaction mixture containing 50 mM phosphate buffer (pH 7), 0.2% guaiacol, 5 mM H_2_O_2_, and 40 μl of plant extract. Absorbance at 470 nm was measured during 3 min and peroxidase activity was calculated using an extinction coefficient of 26.6 mM^−1^·cm^−1^ in three technical replicates. One unit of peroxidase was expressed as the amount of enzyme that causes the formation of 1 mM of tetraguaiacol per minute (Uarrota et al., 2016). We analysed six biological replicates, each containing the pooled material of at least four plants.

#### 4.5.5 Plant hormone measurements

Levels of IAA, SA, ABA, and JA were measured in root material using a UHPLC Q‐Exactive high‐resolution Orbitrap mass spectrometer (Thermo Fisher Scientific) following a cold solvent (modified Bieleski) extraction and centrifugal filtration clean‐up, according to the protocol described in Haeck et al. (2018). ET measurement was performed using gas chromatography (Thermo Finnigan TRACE GC Ultra) according to the procedure described by Singh et al. (2020a). We analysed six biological replicates per treatment, each containing the pooled material of at least four plants.

### 4.6 RT‐qPCR

RNA was extracted from root tissues using the RNeasy Plant Mini Kit (Qiagen) and treated with DNase I (ThermoFisher Scientific). First‐strand cDNA was synthesized using a Tetro cDNA Synthesis Kit (Bioline). All qPCRs were performed using a SensiMix SYBR HI‐ROX kit (Bioline) on three biological and three technical replicates with a CFX Connect Real‐Time PCR Detection System (Bio‐Rad), using the conditions as described in De Kesel et al. (2020). Primers are listed in Table S1. Gene expression levels were normalized using two reference genes (Kyndt et al., 2012b) and the data were statistically analysed using REST 2009 (Pfaffl et al., 2002).

### 4.7 Statistical analysis

Except for differential expression analyses, statistical analyses (analysis of variance [ANOVA], Student's *t* test and post hoc tests applied when appropriate, as indicated in the corresponding figure legends) were performed in SPSS Statistics v. 26.0 and R software v. 4.0.2. The assumptions of normality and homogeneity of the data were checked and found to be fulfilled.

## CONFLICT OF INTEREST

The authors declare that they have no conflict of interests.

## Supporting information


**FIGURE S1** Effect of different concentrations of dehydroascorbate (DHA), namely, 1, 5, 10, 20, and 30 mM, on the shoot and root lengths in rice. Error bars on each column indicate *SE* from eight replicates. Different letters on error bars within a group indicate a statistically significant difference (Duncan’s multiple range test, α = 0.05)

**FIGURE S2** Long‐term effects of dehydroascorbate (DHA) treatment on rice growth and yield. Plant height (cm) of cultivars (a) Kitaake and (b) Nipponbare measured at biweekly intervals during the entire growth cycle of the plants. Effect of DHA on the number of tillers, panicles, and seed weight of (c) Kitaake and (d) Nipponbare, recorded at the time of plant harvest. (e) Kitaake and (f) Nipponbare at 14 weeks after planting. Plants were treated with 20 mM DHA at biweekly intervals during the entire growth cycle. The corresponding nontreated control plants were grown under the same conditions and were mock‐treated with water. Error bars indicate the *SE* from nine plants. Asterisks indicate statistically significant differences with the mock‐treated control plants, Student’s *t* test, *p < 0.05

**FIGURE S3** MapMan visualization showing the differential expression pattern in plant hormone pathways, based on the log_2_ fold changes in dehydroascorbate (DHA) 20 mM at 1 and at 4 days posttreatment (DPT), nematode infected at 4 DPT/3 days postinocualtion (DPI), DHA 20 mM + nematode‐infected at 4 DPT/3 DPI rice roots in comparison with roots of mock‐treated control plants or mock‐treated plus nematode‐infected control plants. Each square in the display represents one rice transcript annotated by MapMan to belong to this category of genes. A transcript is coloured blue if this transcript is induced (log_2_ FC > 0) or red if this transcript is repressed (log_2_ FC < 0)

**FIGURE S4** MapMan visualization showing the differential expression pattern in the phenylpropanoid lignin and lignans and terpenoid pathway, based on the log_2_ fold changes of mRNA levels in dehydroascorbate (DHA) 20 mM at 1 and at 4 days posttreatment (DPT), nematode‐infected at 4 DPT/3 days postinoculation (DPI), DHA 20 mM + nematode‐infected at 4 DPT/3 DPI rice roots in comparison with roots of mock‐treated control plants or mock‐treated plus nematode‐infected control plants. Each square in the display represents one rice transcript annotated by MapMan to belong to this category of genes. A transcript is coloured blue if this transcript is induced (log_2_ FC > 0) or red if this transcript is repressed (log_2_ FC < 0)

**FIGURE S5** Nematode infection experiment using reactive oxygen species (ROS) inhibitors (a) catalase and (b) dimethylthiourea (DMTU). Plants were treated with dehydroascorbate (DHA), catalase or DMTU, alone or in combination with 20 mM DHA. Around 250 second‐stage juveniles of *Meloidogyne graminicola* were inoculated per plant at 1 day posttreatment. Galls, nematodes, and egg‐laying females were recorded 2 weeks after nematode inoculation. Error bars indicate the *SE* of eight replications. The whole experiment was independently repeated twice, providing confirmatory results. Different letters indicate a statistically significant difference, Duncan’s multiple range test, α = 0.05

**FIGURE S6** Relative expression level of *WRKY45* in the shoot and root tissues of *WRKY45*‐RNAi plants in comparison with wild‐type Nipponbare

**TABLE S1** Overview of the target genes used in the study, showing the Rice Genome Locus Number, primer pair used for reverse transcription‐quantitative PCR, and the pathway in which each gene is mainly involved

**TABLE S2** GO terms enriched on dehydroascorbate (DHA) treatment, based on mRNA‐Seq data of: 1, DHA 20 mM vs. control at 1 day posttreatment (DPT); 2, DHA 20 mM vs. control at 4 DPT; 3, nematode inoculation vs. control; 4, DHA20 mM + nematode inoculation vs. control; 5, DHA20 mM + nematode inoculation vs. mock‐treated + nematode‐inoculated plants. GO terms of the significantly differentially expressed gene sets from up‐regulated (Up) and down‐regulated (Down) differentially expressed genes were identified using g:Profiler. CTRL, mock‐treated control plants

**TABLE S3** List of enriched GO terms based on mRNA‐Seq analysis in roots of rice plants treated with 5 or 20 mM dehydroascorbate (DHA) at 1 and 4 days posttreatment (DPT), nematodeinoculated at 3 days postinoculation (DPI) compared to mock‐treated plants, and DHA20 + nematode‐inoculated plants at 3 DPI in comparison with mock‐treated + nematode‐inoculated plants. *p* values are FDR‐adjusted. GO terms highlighted in bold are mainly associated with plant stress responses. MF, molecular function; CC, cellular component; BP, biological processes; CTRL, mock‐treated control plants

**TABLE S4** Detailed MapMan analysis showing the significantly enriched processes based on mRNA‐Seq data of 5 and 20 mM dehydroascorbate (DHA) at 1 and 4 days posttreatment (DPT), nematode‐inoculated at 3 days postinoculation (DPI) compared to mock‐treated plants, and DHA20 + nematode‐inoculated plants at 3 DPI in comparison with mock‐treated + nematode‐inoculated plants. Enriched processes highlighted in bold are mainly associated with plant stress responses. *p* values are FDR adjusted. CTRL, mock‐treated control plants

**TABLE S5** Overview of root expression pattern of genes responsive to (a) ethylene, (b) jasmonic acid, (c) abscisic acid (ABA), (d) indole acetic acid (IAA), (e) WRKY transcription factors, (f) phenylpropanoid, and (g) terpenoid phytoalexin pathway, obtained through MapMan analysis based on mRNA‐Seq data from dehydroascorbate (DHA) 20 mM at 1 and at 4 days posttreatment (DPT), nematode‐infected at 4 DPT/3 days postinoculation (DPI), DHA 20 mM + nematode‐infected at 4 DPT/3 DPI rice roots in comparison with roots of mock‐treated control plants or mock‐treated plus nematode‐infected control plants. The table shows the log_2_ fold change (FC) of the gene expression in treated versus control. Colour scale gradient blue to red indicates highly induced transcript to highly repressed transcripts. Values indicated in bold represent significant differential expression (FDR < 0.05). CTRL, mock‐treated control plants

**TABLE S6** Overview of read numbers per sample during mRNA‐Seq data processing. Samples were four‐way multiplexed, hence the subsample column. The mapping column denotes the number of reads that were uniquely aligned to the genome

**TABLE S7** List of differentially expressed genes (DEGs) for all comparisons

## Data Availability

The data that support the findings of this study are openly available at NCBI https://www.ncbi.nlm.nih.gov/bioproject, as BioProject PRJNA767540.
