# Supplementary material for: Dehydroascorbate induces plant resistance in rice against root‐knot nematode Meloidogyne graminicola
Source: Mol Plant Pathol. 2022 May 19;23(9):1303–19. doi: 10.1111/mpp.13230 (PMC9366072; doi:10.1111/mpp.13230)
Supplement: Supplementary file 1 — FIGURE S1 Effect of different concentrations of dehydroascorbate (DHA), namely, 1, 5, 10, 20, and 30 mM, on the shoot and root lengths in rice. Error bars on each column indicate SE from eight replicates. Different letters on error bars within a group indicate a statistically significant difference (Duncan’s multiple range test, α = 0.05) [file MPP-23-1303-s012.pdf]

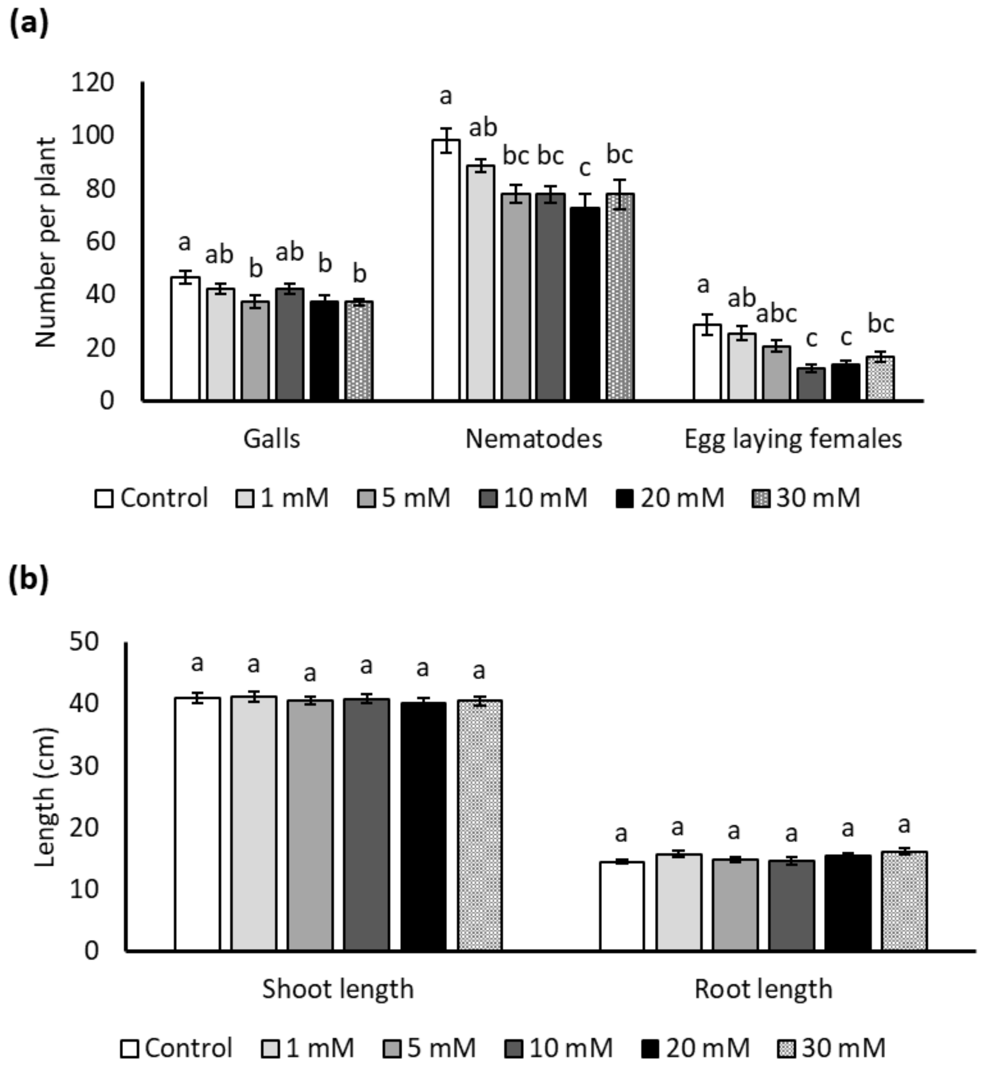

FIGURE S1 Effect of different concentrations of DHA *viz*., 1, 5, 10, 20, and 30 mM on plant susceptibility to *Meloidogyne graminicola*. (a) Effect on galls, nematodes, and reproduction of *M. graminicola*. (b) Effect on the shoot and root lengths in rice. Error bars on each column indicate SE from eight replicates. Different letters on error bars within a group indicate a statistically significant difference (Duncan's multiple range test; α=0.05)
