# Supplementary material for: Dehydroascorbate induces plant resistance in rice against root‐knot nematode Meloidogyne graminicola
Source: Mol Plant Pathol. 2022 May 19;23(9):1303–19. doi: 10.1111/mpp.13230 (PMC9366072; doi:10.1111/mpp.13230)
Supplement: Supplementary file 2 — FIGURE S2 Long‐term effects of dehydroascorbate (DHA) treatment on rice growth and yield. Plant height (cm) of cultivars (a) Kitaake and (b) Nipponbare measured at biweekly intervals during the entire growth cycle of the plants. Effect of DHA on the number of tillers, panicles, and seed weight of (c) Kitaake and (d) Nipponbare, recorded at the time of plant harvest. (e) Kitaake and (f) Nipponbare at 14 weeks after planting. Plants were treated with 20 mM DHA at biweekly intervals during the entire growth cycle. The corresponding nontreated control plants were grown under the same conditions and were mock‐treated with water. Error bars indicate the SE from nine plants. Asterisks indicate statistically significant differences with the mock‐treated control plants, Student’s t test, *p < 0.05 [file MPP-23-1303-s001.pdf]

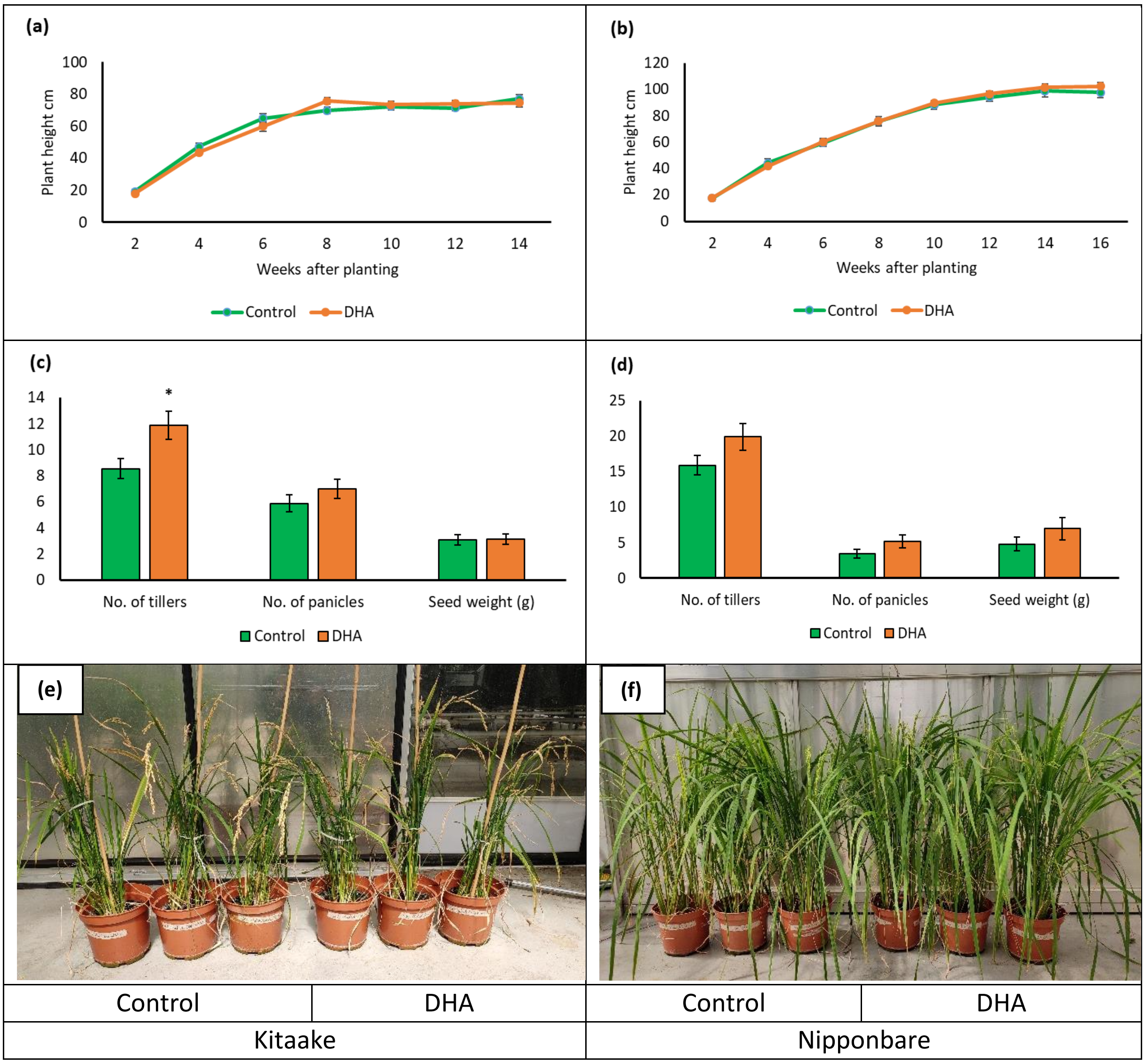

FIGURE S2 The long-term effects of DHA treatment on rice growth and yield. Plant height (cm) of cultivar (a) Kitaake and (b) Nipponbare measured at two-week interval during the entire growth cycle of the plants. Effect of DHA on number of tillers, panicles, and seed weight of plants of (c) Kitaake and (d) Nipponbare, recorded at the time of plant harvest. Plants of (e) Kitaake and (f) Nipponbare at 14 weeks after planting. Plants were treated with 20 mM DHA at every two-week interval during the entire growth cycle. The corresponding non-treated control plants were also grown under the same conditions and were mock-treated with water. Error bars indicate the SE from nine plants. *Asterisks on error bars indicate statistically significant difference with the mock-treated control plants (Student's t-test, * = $p < 0.05$)
