# Supplementary material for: Dehydroascorbate induces plant resistance in rice against root‐knot nematode Meloidogyne graminicola
Source: Mol Plant Pathol. 2022 May 19;23(9):1303–19. doi: 10.1111/mpp.13230 (PMC9366072; doi:10.1111/mpp.13230)
Supplement: Supplementary file 3 — FIGURE S3 MapMan visualization showing the differential expression pattern in plant hormone pathways, based on the log2 fold changes in dehydroascorbate (DHA) 20 mM at 1 and at 4 days posttreatment (DPT), nematode infected at 4 DPT/3 days postinocualtion (DPI), DHA 20 mM + nematode‐infected at 4 DPT/3 DPI rice roots in comparison with roots of mock‐treated control plants or mock‐treated plus nematode‐infected control plants. Each square in the display represents one rice transcript annotated by MapMan to belong to this category of genes. A transcript is coloured blue if this transcript is induced (log2 FC > 0) or red if this transcript is repressed (log2 FC < 0) [file MPP-23-1303-s003.pdf]

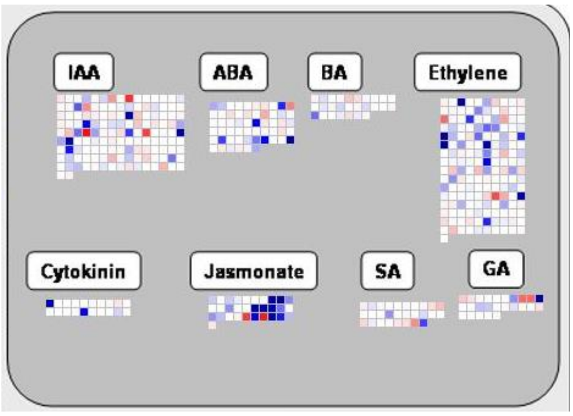

DHA20 v CTRL (1DPT)

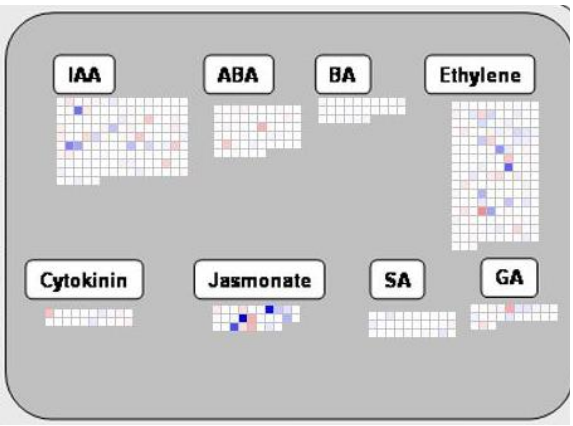

DHA20 v CTRL (4DPT)

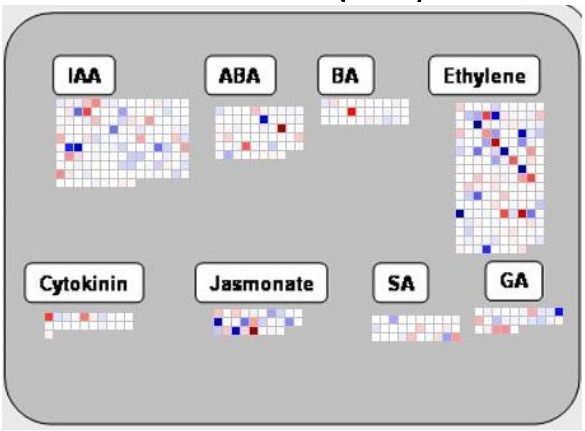

Nematode v CTRL (4DPT/3DPI)

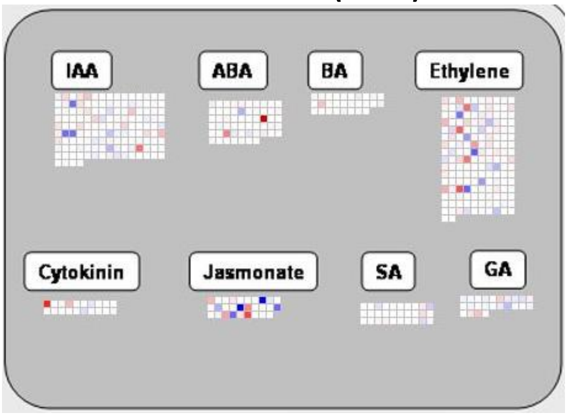

DHA20+Nematode v CTRL (4DPT/3DPI)

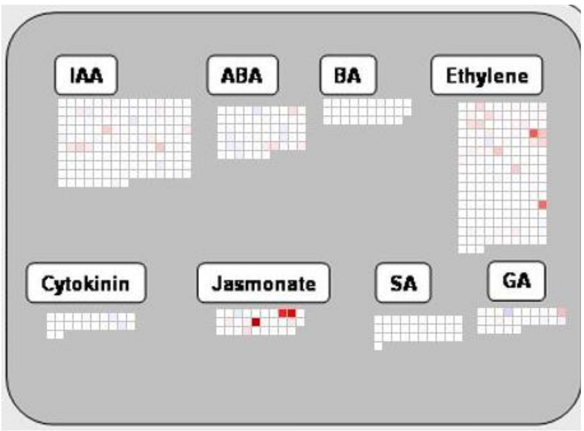

DHA20+Nematode v CTRL+Nematode (4DPT/3DPI)

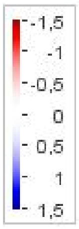

FIGURE S3 Mapman visualization showing the differential expression pattern in plant hormone pathways, based on the Log2 fold changes of mRNA levels, in DHA 20 mM at 1 and at 4 DPT, nematode infected at 4DPT/3DPI, DHA 20 mM + nematode infected at 4DPT/3DPI rice roots in comparison with roots of mock-treated control plants or mock-treated plus nematode infected control plants. Each square in the display represents one rice transcript annotated by MapMan to belong to this category of genes. A transcript is colored blue if this transcript is induced (Log2FC>0) or red if this transcript is repressed (Log2FC<0)
