# Supplementary material for: Dehydroascorbate induces plant resistance in rice against root‐knot nematode Meloidogyne graminicola
Source: Mol Plant Pathol. 2022 May 19;23(9):1303–19. doi: 10.1111/mpp.13230 (PMC9366072; doi:10.1111/mpp.13230)
Supplement: Supplementary file 5 — FIGURE S5 Nematode infection experiment using reactive oxygen species (ROS) inhibitors (a) catalase and (b) dimethylthiourea (DMTU). Plants were treated with dehydroascorbate (DHA), catalase or DMTU, alone or in combination with 20 mM DHA. Around 250 second‐stage juveniles of Meloidogyne graminicola were inoculated per plant at 1 day posttreatment. Galls, nematodes, and egg‐laying females were recorded 2 weeks after nematode inoculation. Error bars indicate the SE of eight replications. The whole experiment was independently repeated twice, providing confirmatory results. Different letters indicate a statistically significant difference, Duncan’s multiple range test, α = 0.05 [file MPP-23-1303-s004.pdf]

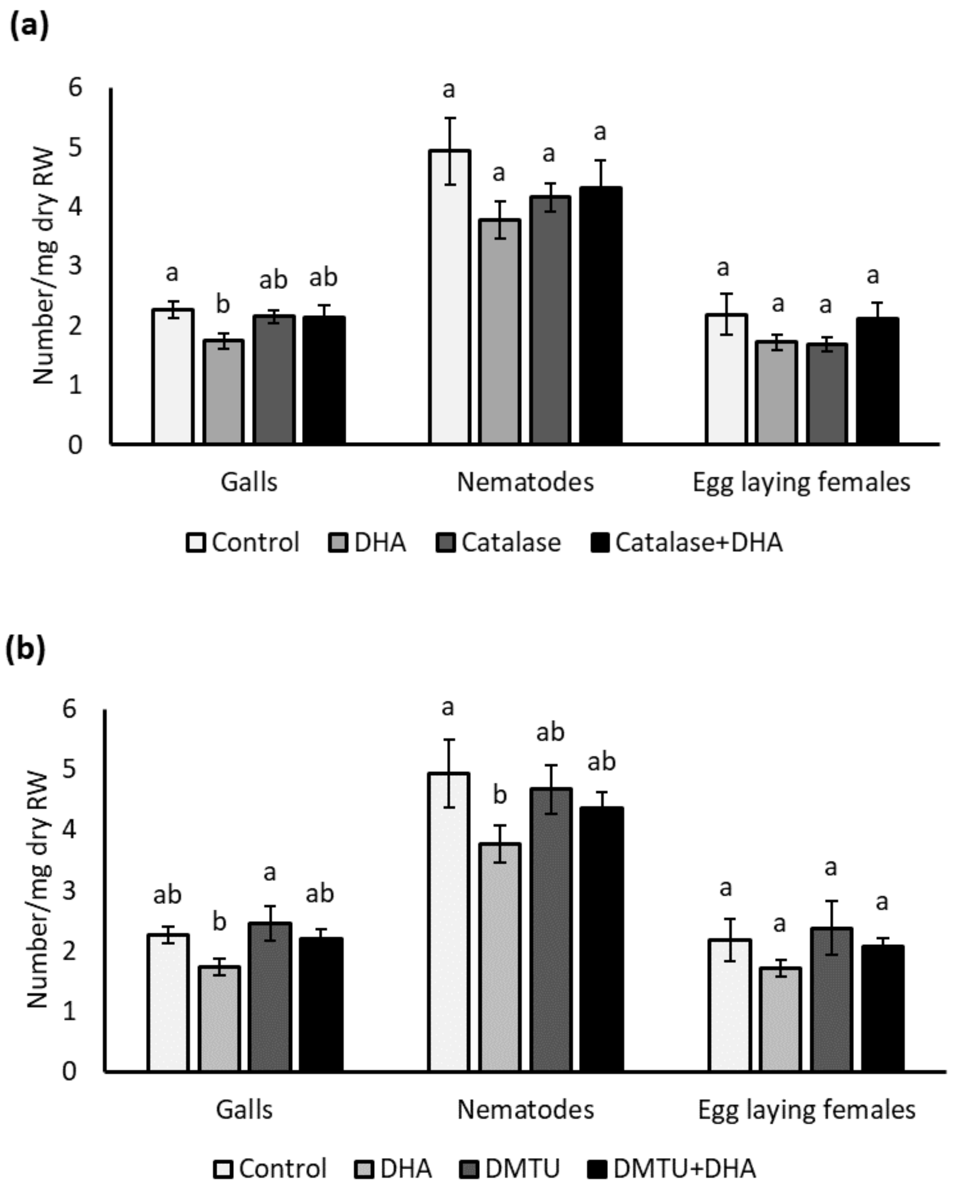

FIGURE S5 Nematode infection experiment using ROS inhibitors (a) catalase, and (b) dimethylthiourea (DMTU). Plants were treated with either DHA, catalase or DMTU, alone or in combination with 20 mM DHA. Around 250 second-stage juveniles of *Meloidogyne graminicola* were inoculated per plant at 1 DPT. Observations on galls, nematodes, and egg laying females were recorded two-week post nematode inoculation. Error bars on each column indicate the SE of eight replications. The whole experiment was independently repeated twice, providing confirmatory results. Different letters on error bars within a group indicate a statistically significant difference (Duncan's multiple range test; α=0.05)
