# Supplementary material for: Dehydroascorbate induces plant resistance in rice against root‐knot nematode Meloidogyne graminicola
Source: Mol Plant Pathol. 2022 May 19;23(9):1303–19. doi: 10.1111/mpp.13230 (PMC9366072; doi:10.1111/mpp.13230)
Supplement: Supplementary file 6 — FIGURE S6 Relative expression level of WRKY45 in the shoot and root tissues of WRKY45‐RNAi plants in comparison with wild‐type Nipponbare [file MPP-23-1303-s007.docx]

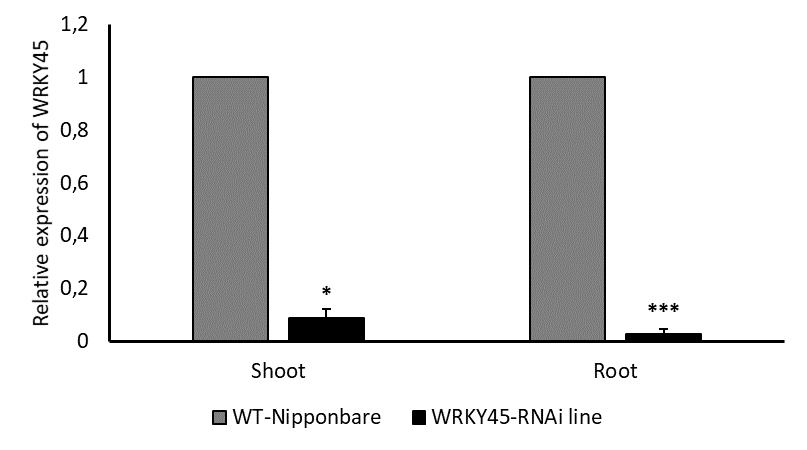


**FIGURE S6** Relative expression level of *WRKY45* in the shoot and root tissues of WRKY45-RNAi plants in comparison with wild type Nipponbare. Expression levels were determined by qRT-PCR and were expressed relative to wild-type Nipponbare plants. qRT-PCRs were performed with two technical replicates on three biologically independent replicates. Error bars indicate the 95% confidence interval as calculated by Rest2009 software. Gene expression levels were normalized using two internal reference genes, OsEXP and OsEXPNarsi. *Asterisks indicate significant differential expression in comparison with the control plants (REST-analysis; * = p < 0.05, ** = p < 0.01, *** = p < 0.001)
