# Supplementary material for: Dehydroascorbate induces plant resistance in rice against root‐knot nematode Meloidogyne graminicola
Source: Mol Plant Pathol. 2022 May 19;23(9):1303–19. doi: 10.1111/mpp.13230 (PMC9366072; doi:10.1111/mpp.13230)
Supplement: Supplementary file 7 — TABLE S1 Overview of the target genes used in the study, showing the Rice Genome Locus Number, primer pair used for reverse transcription‐quantitative PCR, and the pathway in which each gene is mainly involved [file MPP-23-1303-s011.docx]

**TABLE S1** Overview of the target genes used in the study, showing the Rice Genome Locus Number, primer pair used for qRT-PCR and the pathway in which each gene is mainly involved.

| **Genes** | **Locus no.** | **Forward primer** | **Reverse primer** | **Mainly involved in** |
| --- | --- | --- | --- | --- |
| OsWRKY45 | LOC_Os05g25770 | AATTCGGTGGTCGTCAAGAA | AAGTAGGCCTTTGGGTGCTT | SA responsive |
| OsPR1a | LOC_Os07g0418500 | TCGTATGCTATGCTACGTGTTT | CACTAAGCAAATACGGCTGACA | SA responsive |
| OsPR1b | LOC_Os01g28450 | GGCAACTTCGTCGGACAGA | CCGTGGACCTGTTTACATTTT | SA responsive |
| OsEXP | LOC_Os03g27010 | TGTGAGCAGCTTCTCGTTTG | TGTTGTTGCCTGTGAGATCG | Reference gene |
| OsEXPNarcai | LOC_Os07g02340 | AGGAACATGGAGAAGAACAAGG | CAGAGGTGGTGCAGATGAAA | Reference gene |
