# Supplementary material for: Dehydroascorbate induces plant resistance in rice against root‐knot nematode Meloidogyne graminicola
Source: Mol Plant Pathol. 2022 May 19;23(9):1303–19. doi: 10.1111/mpp.13230 (PMC9366072; doi:10.1111/mpp.13230)
Supplement: Supplementary file 8 — TABLE S2 GO terms enriched on dehydroascorbate (DHA) treatment, based on mRNA‐Seq data of: 1, DHA 20 mM vs. control at 1 day posttreatment (DPT); 2, DHA 20 mM vs. control at 4 DPT; 3, nematode inoculation vs. control; 4, DHA20 mM + nematode inoculation vs. control; 5, DHA20 mM + nematode inoculation vs. mock‐treated + nematode‐inoculated plants. GO terms of the significantly differentially expressed gene sets from up‐regulated (Up) and down‐regulated (Down) differentially expressed genes were identified using g:Profiler. CTRL, mock‐treated control plants [file MPP-23-1303-s002.docx]

**TABLE S2** Overview of GO terms enriched upon DHA treatment, based on mRNA-seq data of 1. DHA 20 mM vs Control at 1 DPT, 2. DHA 20 mM vs control at 4 DPT, 3. Nematode inoculation vs control, 4. DHA20 mM+Nematode inoculation vs control, 5. DHA20 mM+Nematode inoculation vs mock treated+Nematode inoculated plants. Gene Ontology terms with enriched molecular functions and associated biological pathways of the significantly differentially expressed gene sets from upregulated (Up) and downregulated (Down) set of DEGs were identified using g:Profiler tool. CTRL: mock-treated control plants

|  | DHA20 vs CTRL (1DPT) | DHA20 vs CTRL (4DPT) | Nematode vs CTRL (4DPT/3DPI) | DHA20+Nematode  vs CTRL (4DPT/3DPI) | DHA20+Nematode  vs CTRL+Nematode (4DPT/3DPI) |
| --- | --- | --- | --- | --- | --- |
| Response to oxidative stress | Up | Up | Mixed | Up | Down |
| Defense response | Up | Up | Up | Up | - |
| Response to chemical | Up | Up | Mixed | - | - |
| Response to wounding | Up | - | - | - | Down |
| Response to stress | - | Up | - | Up | Down |
| Response to toxic substance | - | - | Up | Up | Down |
| Response to biotic stimulus | - | - | Mixed | - | - |
| Response to heat | - | - | Down | - | Up |
| Response to temperature | - | - | Down | - | Up |
| Cellular response to hormone stimulus | - | - | Down | - | - |
| Secondary metabolite biosynthesis and metabolism | Up | - | - | - | - |
| Terpenoid biosynthetic process | - | Up | - | - | Down |
| Terpenoid metabolic process | Up |  | - | - | Down |
| Diterpenoid biosynthetic process | Up | Up | - | - | Down |
| Diterpenoid metabolic process | Up | Up | - | - | Down |
