# Supplementary material for: Dehydroascorbate induces plant resistance in rice against root‐knot nematode Meloidogyne graminicola
Source: Mol Plant Pathol. 2022 May 19;23(9):1303–19. doi: 10.1111/mpp.13230 (PMC9366072; doi:10.1111/mpp.13230)
Supplement: Supplementary file 9 — TABLE S3 List of enriched GO terms based on mRNA‐Seq analysis in roots of rice plants treated with 5 or 20 mM dehydroascorbate (DHA) at 1 and 4 days posttreatment (DPT), nematodeinoculated at 3 days postinoculation (DPI) compared to mock‐treated plants, and DHA20 + nematode‐inoculated plants at 3 DPI in comparison with mock‐treated + nematode‐inoculated plants. p values are FDR‐adjusted. GO terms highlighted in bold are mainly associated with plant stress responses. MF, molecular function; CC, cellular component; BP, biological processes; CTRL, mock‐treated control plants [file MPP-23-1303-s006.docx]

**TABLE S3** List of enriched gene ontology (GO) terms based on mRNA-seq analysis in roots of rice plants treated with 5 or 20 mM DHA at 1 and 4 days post treatment, nematode inoculated at 3 days post inoculation compared to mock treated plants and DHA20+nematode inoculated plants at 3 days post nematode inoculation in comparison with mock treated+nematode inoculated plants. P-values are FDR-adjusted. GO terms highlighted in bold are mainly associated with plant stress responses. (MF: molecular function, CC: cellular component, BP: biological processes). CTRL: mock-treated control plants.

| **Upregulated set of DEGs: DHA5 v CTRL and DHA20 v CTRL at 1 DPT** | | |
| --- | --- | --- |
| **Common in DHA5 v CTRL and DHA20 v CTRL at 1 DPT** | | |
| **GO term** | **Function** | **p** |
| GO:BP | Aromatic amino acid family biosynthetic process | <0.001 |
| GO:BP | Aromatic amino acid family metabolic process | <0.001 |
| GO:BP | Organic acid biosynthetic process | 0.004 |
| GO:BP | Tryptophan biosynthetic process | 0.005 |
| GO:BP | Indolalkylamine biosynthetic process | 0.005 |
| GO:BP | Cellular amino acid metabolic process | 0.005 |
| GO:BP | Alpha-amino acid metabolic process | 0.007 |
| GO:BP | Amine biosynthetic process | 0.007 |
| GO:BP | Cellular biogenic amine biosynthetic process | 0.007 |
| GO:BP | Carboxylic acid biosynthetic process | 0.009 |
| GO:BP | Amine metabolic process | 0.010 |
| GO:BP | Cellular amino acid biosynthetic process | 0.020 |
| GO:BP | Tryptophan metabolic process | 0.040 |
| GO:BP | Indolalkylamine metabolic process | 0.040 |
| GO:CC | Anchored component of membrane | 0.004 |
| GO:CC | Intrinsic component of plasma membrane | 0.004 |
| GO:CC | Plasma membrane | 0.010 |
| GO:CC | Anchored component of plasma membrane | 0.020 |
| KEGG | **Phenylalanine, tyrosine and tryptophan biosynthesis** | <0.001 |
|  |  |  |
| **DHA5 v CTRL 1 DPT** | | |
| Nil |  |  |
|  |  |  |
| **DHA20 v CTRL 1 DPT** | | |
| GO:MF | **Catalytic activity** | <0.001 |
| GO:MF | **Oxidoreductase activity** | <0.001 |
| GO:MF | Ion binding | <0.001 |
| GO:MF | Heme binding | <0.001 |
| GO:MF | Tetrapyrrole binding | <0.001 |
| GO:MF | Unfolded protein binding | 0.003 |
| GO:MF | Lyase activity | 0.008 |
| GO:MF | **Oxidoreductase activity**, acting on the CH-OH group of donors, NAD or NADP as acceptor | 0.010 |
| GO:MF | Vitamin B6 binding | 0.020 |
| GO:MF | Pyridoxal phosphate binding | 0.020 |
| GO:MF | Manganese ion binding | 0.020 |
| GO:MF | Structural molecule activity | 0.020 |
| GO:MF | **Oxidoreductase activity**, acting on CH-OH group of donors | 0.020 |
| GO:MF | Small molecule binding | 0.040 |
| GO:MF | Structural constituent of ribosome | 0.040 |
| GO:BP | Small molecule metabolic process | <0.001 |
| GO:BP | Organic acid metabolic process | <0.001 |
| GO:BP | Carboxylic acid metabolic process | <0.001 |
| GO:BP | Oxoacid metabolic process | <0.001 |
| GO:BP | Organic acid biosynthetic process | <0.001 |
| GO:BP | Carboxylic acid biosynthetic process | <0.001 |
| GO:BP | Small molecule biosynthetic process | <0.001 |
| GO:BP | **Diterpenoid metabolic process** | <0.001 |
| GO:BP | **Defense response** | <0.001 |
| GO:BP | Carbohydrate derivative metabolic process | <0.001 |
| GO:BP | **Diterpenoid biosynthetic process** | <0.001 |
| GO:BP | **Oxidation-reduction process** | <0.001 |
| GO:BP | **Secondary metabolite biosynthetic process** | <0.001 |
| GO:BP | Protein folding | 0.001 |
| GO:BP | **Organonitrogen compound biosynthetic process** | 0.003 |
| GO:BP | Monocarboxylic acid metabolic process | 0.003 |
| GO:BP | **Secondary metabolic process** | 0.003 |
| GO:BP | Aminoglycan metabolic process | 0.004 |
| GO:BP | Nucleobase-containing small molecule metabolic process | 0.005 |
| GO:BP | Aromatic amino acid family metabolic process | 0.010 |
| GO:BP | Cellular amino acid biosynthetic process | 0.010 |
| GO:BP | **Response to wounding** | 0.020 |
| GO:BP | **Organonitrogen compound metabolic process** | 0.020 |
| GO:BP | Amino sugar catabolic process | 0.020 |
| GO:BP | Aminoglycan catabolic process | 0.020 |
| GO:BP | Glucosamine-containing compound catabolic process | 0.020 |
| GO:BP | Chitin catabolic process | 0.020 |
| GO:BP | Chitin metabolic process | 0.020 |
| GO:BP | Alpha-amino acid metabolic process | 0.020 |
| GO:BP | **Diterpene phytoalexin biosynthetic process** | 0.030 |
| GO:BP | **Diterpene phytoalexin metabolic process** | 0.030 |
| GO:BP | **Toxin biosynthetic process** | 0.030 |
| GO:BP | **Phytoalexin biosynthetic process** | 0.030 |
| GO:BP | **Toxin metabolic process** | 0.030 |
| GO:BP | **Phytoalexin metabolic process** | 0.030 |
| GO:BP | **Response to stress** | 0.030 |
| GO:BP | **Terpenoid metabolic process** | 0.040 |
| GO:BP | Cellular amino acid metabolic process | 0.040 |
| GO:BP | **Response to fungus** | 0.050 |
| GO:BP | Oxylipin metabolic process | 0.050 |
| GO:BP | Oxylipin biosynthetic process | 0.050 |
| GO:CC | Cytosol | <0.001 |
| GO:CC | Cytosolic ribosome | <0.001 |
| GO:CC | Cytoplasm | <0.001 |
| GO:CC | Ribosomal subunit | <0.001 |
| GO:CC | Cytosolic large ribosomal subunit | 0.002 |
| GO:CC | Golgi apparatus | 0.005 |
| GO:CC | Vesicle coat | 0.010 |
| GO:CC | Cytosolic small ribosomal subunit | 0.020 |
| GO:CC | Ribosome | 0.030 |
| GO:CC | Coated vesicle membrane | 0.040 |
| GO:CC | Extracellular region | 0.040 |
| GO:CC | Organelle membrane | 0.040 |
| GO:CC | Endomembrane system | 0.050 |
| GO:CC | Coated vesicle | 0.050 |
| KEGG | **Diterpenoid biosynthesis** | <0.001 |
| KEGG | Alpha-Linolenic acid metabolism | <0.001 |
| KEGG | **Biosynthesis of secondary metabolites** | <0.001 |
| KEGG | Biosynthesis of amino acids | 0.001 |
| KEGG | Amino sugar and nucleotide sugar metabolism | 0.010 |

| **Downregulated Set of DEGs: DHA5 v CTRL and DHA20 v CTRL at 1 DPT** | | |
| --- | --- | --- |
| **Common in DHA5 v CTRL and DHA20 v CTRL at 1 DPT** | | |
| GO:MF | **Peroxidase activity** | <0.001 |
| GO:MF | **Oxidoreductase activity** | <0.001 |
| GO:MF | **Antioxidant activity** | <0.001 |
| GO:MF | Water channel activity | <0.001 |
| GO:MF | Water transmembrane transporter activity | <0.001 |
| GO:MF | Heme binding | 0.003 |
| GO:MF | Tetrapyrrole binding | 0.003 |
| GO:MF | Inorganic molecular entity transmembrane transporter activity | 0.004 |
| GO:MF | Transmembrane transporter activity | 0.005 |
| GO:MF | Transporter activity | 0.007 |
| GO:MF | Channel activity | 0.010 |
| GO:MF | Passive transmembrane transporter activity | 0.020 |
| GO:BP | **Hydrogen peroxide catabolic process** | <0.001 |
| GO:BP | **Hydrogen peroxide metabolic process** | <0.001 |
| GO:BP | **Reactive oxygen species metabolic process** | <0.001 |
| GO:BP | **Cellular oxidant detoxification** | <0.001 |
| GO:BP | **Cellular response to toxic substance** | <0.001 |
| GO:BP | **Cellular detoxification** | <0.001 |
| GO:BP | **Detoxification** | <0.001 |
| GO:BP | **Response to toxic substance** | <0.001 |
| GO:BP | Water transport | <0.001 |
| GO:BP | Fluid transport | <0.001 |
| GO:BP | **Response to oxidative stress** | <0.001 |
| GO:BP | Transmembrane transport | 0.020 |
| GO:BP | **Response to chemical** | 0.030 |
| GO:BP | Inorganic anion transport | 0.040 |
| GO:CC | Cell periphery | <0.001 |
| GO:CC | Cell wall | <0.001 |
| GO:CC | External encapsulating structure | <0.001 |
| GO:CC | Extracellular region | <0.001 |
| GO:CC | Plant-type cell wall | <0.001 |
| GO:CC | Anchoring junction | <0.001 |
| GO:CC | Symplast | <0.001 |
| GO:CC | Cell junction | <0.001 |
| GO:CC | Plasmodesma | <0.001 |
| GO:CC | Cell-cell junction | <0.001 |
| GO:CC | Vacuole | <0.001 |
| KEGG | **Phenylpropanoid biosynthesis** | <0.001 |
| KEGG | **Biosynthesis of secondary metabolites** | 0.006 |
| KEGG | **Metabolic pathways** | 0.006 |
|  |  |  |
| **DHA5 v CTRL 1 DPT** | | |
| GO:MF | L-malate dehydrogenase activity | 0.050 |
| GO:CC | Extracellular region | 0.001 |
| GO:CC | Anchored component of membrane | 0.008 |
| KEGG | Citrate cycle (TCA cycle) | 0.010 |
| KEGG | Glyoxylate and dicarboxylate metabolism | 0.030 |
| KEGG | Pyruvate metabolism | 0.050 |
|  |  |  |
| **DHA20 v CTRL 1 DPT** | | |
| GO:MF | Cation binding | 0.050 |
| GO:BP | Protein ubiquitination | 0.020 |
| GO:BP | Protein modification by small protein conjugation | 0.050 |
| KEGG | Circadian rhythm - plant | 0.010 |
| KEGG | Basal transcription factors | 0.050 |

| **Upregulated set of DEGs: : DHA5 v CTRL and DHA20 mM v CTRL at 4 DPT** | | |
| --- | --- | --- |
| **Common in DHA5 v CTRL and DHA20 mM v CTRL at 4 DPT** | | |
| Nil |  |  |
|  |  |  |
| **DHA5 v CTRL 4 DPT** | | |
| GO:MF | Unfolded protein binding | 0.009 |
| GO:MF | Misfolded protein binding | 0.010 |
| GO:MF | Protein folding chaperone | 0.020 |
| GO:BP | **Cellular response to heat** | 0.002 |
| GO:BP | **Response to heat** | 0.020 |
| KEGG | Endocytosis | 0.010 |
| KEGG | Spliceosome | 0.020 |
| KEGG | Protein processing in endoplasmic reticulum | 0.030 |
|  |  |  |
| **DHA20 v CTRL 4 DPT** | | |
| GO:MF | **oxidoreductase activity** | <0.001 |
| GO:MF | Heme binding | <0.001 |
| GO:MF | Tetrapyrrole binding | <0.001 |
| GO:MF | Metal ion binding | <0.001 |
| GO:MF | Cation binding | <0.001 |
| GO:MF | Hydrolase activity, hydrolyzing O-glycosyl compounds | <0.001 |
| GO:MF | Transition metal ion binding | <0.001 |
| GO:MF | Hydrolase activity, acting on glycosyl bonds | <0.001 |
| GO:MF | **Peroxidase activity** | <0.001 |
| GO:MF | **Oxidoreductase activity, acting on peroxide as acceptor** | <0.001 |
| GO:MF | Manganese ion binding | <0.001 |
| GO:MF | **Antioxidant activity** | 0.002 |
| GO:MF | Nutrient reservoir activity | 0.004 |
| GO:MF | Iron ion binding | 0.004 |
| GO:MF | **Abscisic acid binding** | 0.004 |
| GO:MF | **Isoprenoid binding** | 0.005 |
| GO:MF | **Hormone binding** | 0.010 |
| GO:MF | Protein phosphatase inhibitor activity | 0.010 |
| GO:MF | Phosphatase inhibitor activity | 0.010 |
| GO:MF | Alcohol binding | 0.010 |
| GO:MF | **Catalytic activity** | 0.020 |
| GO:MF | **Oxidoreductase activity, acting on CH-OH group of donors** | 0.030 |
| GO:MF | **Oxidoreductase activity, acting on paired donors, with incorporation or reduction of molecular oxygen** | 0.040 |
| GO:BP | **Defense response** | <0.001 |
| GO:BP | **Oxidation-reduction process** | <0.001 |
| GO:BP | **Diterpenoid metabolic process** | <0.001 |
| GO:BP | **Response to stress** | <0.001 |
| GO:BP | **Diterpenoid biosynthetic process** | <0.001 |
| GO:BP | **Cellular detoxification** | <0.001 |
| GO:BP | **Cellular response to toxic substance** | <0.001 |
| GO:BP | **Terpenoid metabolic process** | <0.001 |
| GO:BP | **Detoxification** | <0.001 |
| GO:BP | **Response to toxic substance** | <0.001 |
| GO:BP | **Response to stimulus** | <0.001 |
| GO:BP | **Terpenoid biosynthetic process** | 0.001 |
| GO:BP | **Isoprenoid metabolic process** | 0.005 |
| GO:BP | **Hydrogen peroxide catabolic process** | 0.006 |
| GO:BP | **Hydrogen peroxide metabolic process** | 0.008 |
| GO:BP | **Cellular oxidant detoxification** | 0.010 |
| GO:BP | **Isoprenoid biosynthetic process** | 0.010 |
| GO:BP | Regulation of protein serine/threonine phosphatase activity | 0.010 |
| GO:BP | **Cellular response to chemical stimulus** | 0.020 |
| GO:BP | Carbohydrate metabolic process | 0.0300 |
| GO:BP | **Response to oxidative stress** | 0.030 |
| GO:BP | **Reactive oxygen species metabolic process** | 0.030 |
| GO:CC | Extracellular region | <0.001 |
| GO:CC | Anchored component of plasma membrane | <0.001 |
| GO:CC | Anchored component of membrane | <0.001 |
| GO:CC | Intrinsic component of plasma membrane | <0.001 |
| GO:CC | Cell wall | 0.005 |
| GO:CC | External encapsulating structure | 0.005 |
| GO:CC | Apoplast | 0.008 |
| GO:CC | Extracellular space | 0.030 |
| KEGG | **Diterpenoid biosynthesis** | <0.001 |
| KEGG | **Biosynthesis of secondary metabolites** | <0.001 |
| KEGG | Alpha-Linolenic acid metabolism | 0.002 |

| **Dodnregulated set of DEGs: DHA5 v CTRL and DHA20 mM v CTRL at 4 DPT** | | |
| --- | --- | --- |
|  |  |  |
| **Common in DHA5 v CTRL and DHA20 mM v CTRL at 4 DPT** | | |
| Nil |  |  |
|  |  |  |
| **DHA20 v CTRL 4 DPT** | | |
| GO:MF | **Dioxygenase activity** | 0.010 |
| GO:MF | **Peroxidase activity** | 0.010 |
| GO:MF | **Oxidoreductase activity, acting on peroxide as acceptor** | 0.010 |
| GO:MF | **Antioxidant activity** | 0.020 |
| KEGG | Stilbenoid, diarylheptanoid and gingerol biosynthesis | 0.010 |
|  |  |  |
| **DHA20 v CTRL 4 DPT** | | |
| Nil |  |  |

| **Upregulated set of DEGs: DHA 20 v CTRL at 1 and 4 DPT** | | |
| --- | --- | --- |
| **Common in 1 and 4 DPT DHA20 v CTRL** | | |
| GO:MF | **Oxidoreductase activity** | 7.70×10^-9^ |
| GO:MF | Heme binding | 1.29×10^-9^ |
| GO:MF | Tetrapyrrole binding | 2.33×10^-7^ |
| GO:MF | Metal ion binding | 1.24×10^-5^ |
| GO:MF | Cation binding | 1.31×10^-5^ |
| GO:MF | Manganese ion binding | 1.15×10^-4^ |
| GO:MF | Transition metal ion binding | 4.61×10^-4^ |
| GO:MF | Nutrient reservoir activity | 6.74×10^-4^ |
| GO:MF | **Abscisic acid binding** | 0.001 |
| GO:MF | **Isoprenoid binding** | 0.001 |
| GO:MF | Protein phosphatase inhibitor activity | 0.003 |
| GO:MF | **Hormone binding** | 0.004 |
| GO:MF | Alcohol binding | 0.004 |
| GO:MF | Phosphatase inhibitor activity | 0.004 |
| GO:MF | Hydrolase activity, hydrolyzing O-glycosyl compounds | 0.006 |
| GO:MF | **Peroxidase activity** | 0.006 |
| GO:MF | **Oxidoreductase activity, acting on peroxide as acceptor** | 0.006 |
| GO:MF | Ion binding | 0.007 |
| GO:MF | Iron ion binding | 0.008 |
| GO:MF | **Antioxidant activity** | 0.010 |
| GO:MF | **Oxidoreductase activity, acting on paired donors, with incorporation or reduction of molecular oxygen** | 0.010 |
| GO:MF | Monocarboxylic acid binding | 0.010 |
| GO:MF | Hydrolase activity, acting on glycosyl bonds | 0.010 |
| GO:MF | Protein phosphatase regulator activity | 0.050 |
| GO:BP | **Defense response** | 2.31×10^-7^ |
| GO:BP | **Response to stress** | 2.70×10^-6^ |
| GO:BP | **Diterpenoid metabolic process** | 2.88×10^-6^ |
| GO:BP | **Oxidation-reduction process** | 8.69×10^-6^ |
| GO:BP | **Diterpenoid biosynthetic process** | 4.74×10^-5^ |
| GO:BP | **Terpenoid metabolic process** | 3.08×10^-4^ |
| GO:BP | **Response to stimulus** | 0.001 |
| GO:BP | **Cellular detoxification** | 0.001 |
| GO:BP | **Cellular response to toxic substance** | 0.001 |
| GO:BP | **Terpenoid biosynthetic process** | 0.002 |
| GO:BP | **Detoxification** | 0.002 |
| GO:BP | **Response to toxic substance** | 0.002 |
| GO:BP | Regulation of protein serine/threonine phosphatase activity | 0.003 |
| GO:BP | **Isoprenoid metabolic process** | 0.003 |
| GO:BP | Negative regulation of hydrolase activity | 0.008 |
| GO:BP | **Isoprenoid biosynthetic process** | 0.010 |
| GO:BP | Negative regulation of protein dephosphorylation | 0.010 |
| GO:BP | Negative regulation of phosphoprotein phosphatase activity | 0.010 |
| GO:BP | Negative regulation of dephosphorylation | 0.020 |
| GO:BP | Negative regulation of phosphatase activity | 0.020 |
| GO:BP | **Cellular response to chemical stimulus** | 0.030 |
| GO:CC | Extracellular region | 1.22×10^-5^ |
| GO:CC | Anchored component of plasma membrane | 3.88×10^-4^ |
| GO:CC | Anchored component of membrane | 6.08×10^-4^ |
| GO:CC | Intrinsic component of plasma membrane | 0.008 |
| GO:CC | Apoplast | 0.010 |
| GO:CC | Cell wall | 0.020 |
| GO:CC | External encapsulating structure | 0.020 |
| KEGG | **Biosynthesis of secondary metabolites** | 5.86×10^-5^ |
| KEGG | **Diterpenoid biosynthesis** | 1.58×10^-7^ |
| KEGG | Alpha-Linolenic acid metabolism | 6.21×10^-4^ |
|  |  |  |
| **1 DPT DHA20 v CTRL** | | |
| GO:MF | **Catalytic activity** | 1.13×10^-9^ |
| GO:MF | Lyase activity | 0.001 |
| GO:MF | Unfolded protein binding | 0.002 |
| GO:MF | Small molecule binding | 0.008 |
| GO:MF | **Oxidoreductase activity** | 0.010 |
| GO:MF | Anion binding | 0.030 |
| GO:MF | Vitamin B6 binding | 0.030 |
| GO:MF | Pyridoxal phosphate binding | 0.030 |
| GO:MF | Ion binding | 0.040 |
| GO:BP | Small molecule metabolic process | 2.10×10^-15^ |
| GO:BP | Organic acid metabolic process | 5,81×10^-14^ |
| GO:BP | Aromatic amino acid family metabolic process | 1.53×10^-12^ |
| GO:BP | Carboxylic acid metabolic process | 5.09×10^-10^ |
| GO:BP | Oxoacid metabolic process | 7.46×10^-10^ |
| GO:BP | Aromatic amino acid family biosynthetic process | 7.99×10^-9^ |
| GO:BP | Organic acid biosynthetic process | 1.43×10^-8^ |
| GO:BP | Carboxylic acid biosynthetic process | 2.58×10^-8^ |
| GO:BP | Small molecule biosynthetic process | 2.62×10^-6^ |
| GO:BP | Cellular amino acid metabolic process | 1.56×10^-5^ |
| GO:BP | Alpha-amino acid metabolic process | 2.24×10^-5^ |
| GO:BP | Cellular amino acid biosynthetic process | 3.28×10^-5^ |
| GO:BP | **Organonitrogen compound biosynthetic process** | 1.59×10^-4^ |
| GO:BP | **Response to wounding** | 0.001 |
| GO:BP | Indole-containing compound biosynthetic process | 0.001 |
| GO:BP | Protein folding | 0.002 |
| GO:BP | Alpha-amino acid biosynthetic process | 0.002 |
| GO:BP | Tryptophan metabolic process | 0.003 |
| GO:BP | Indolalkylamine metabolic process | 0.003 |
| GO:BP | Indolalkylamine biosynthetic process | 0.006 |
| GO:BP | Tryptophan biosynthetic process | 0.006 |
| GO:BP | Chorismate metabolic process | 0.006 |
| GO:BP | Monocarboxylic acid metabolic process | 0.008 |
| GO:BP | Chorismate biosynthetic process | 0.009 |
| GO:BP | **Organonitrogen compound metabolic process** | 0.009 |
| GO:BP | **Secondary metabolite biosynthetic process** | 0.009 |
| GO:BP | Carbohydrate derivative metabolic process | 0.020 |
| GO:BP | Cellular biogenic amine metabolic process | 0.020 |
| GO:BP | Cellular amine metabolic process | 0.030 |
| GO:BP | **Secondary metabolic process** | 0.030 |
| GO:BP | Amine metabolic process | 0.040 |
| GO:CC | Cytosol | 8.68×10^-9^ |
| GO:CC | Cytoplasm | 6.34×10^-8^ |
| GO:CC | Cytosolic ribosome | 5.10×10^-6^ |
| GO:CC | Golgi apparatus | 3.77×10^-4^ |
| GO:CC | Ribosomal subunit | 0.002 |
| GO:CC | Cytoplasmic vesicle | 0.003 |
| GO:CC | Intracellular vesicle | 0.004 |
| GO:CC | Endomembrane system | 0.004 |
| GO:CC | Vesicle coat | 0.005 |
| GO:CC | Cytosolic large ribosomal subunit | 0.006 |
| GO:CC | Cytoplasmic vesicle membrane | 0.009 |
| GO:CC | Golgi apparatus subcompartment | 0.010 |
| GO:CC | Vesicle membrane | 0.010 |
| GO:CC | Vesicle | 0.010 |
| GO:CC | Coated vesicle membrane | 0.020 |
| GO:CC | Coated membrane | 0.030 |
| GO:CC | Membrane coat | 0.030 |
| GO:CC | Coated vesicle | 0.030 |
| GO:CC | Cytosolic small ribosomal subunit | 0.040 |
| GO:CC | Golgi-associated vesicle membrane | 0.040 |
| KEGG | **Phenylalanine, tyrosine and tryptophan biosynthesis** | 3.08×10^-5^ |
| KEGG | Biosynthesis of amino acids | 3.10×10^-4^ |
| KEGG | Amino sugar and nucleotide sugar metabolism | 0.010 |
|  |  |  |
| **4 DPT DHA20 v CTRL** | | |
| GO:CC | Extracellular region | 0.040 |

| **Downregulated set of DEGs: DHA 20 v CTRL at 1 and 4 DPT** | | |
| --- | --- | --- |
|  |  |  |
| **Common in 1 and 4 DPT DHA20 v CTRL** | | |
| GO:MF | **Heat shock protein binding** | 0.030 |
| KEGG | Protein processing in endoplasmic reticulum | 0.001 |
|  |  |  |
| **1 DPT DHA20 v CTRL** | | |
| GO:MF | Cation binding | 0.003 |
| GO:MF | Metal ion binding | 0.003 |
| GO:MF | Water transmembrane transporter activity | 0.010 |
| GO:MF | Water channel activity | 0.010 |
| GO:CC | Plant-type cell wall | 0.0007 |
| GO:CC | Cell wall | 0.010 |
| GO:CC | External encapsulating structure | 0.010 |
| KEGG | Circadian rhythm - plant | 0.010 |
|  |  |  |
| **4 DPT DHA20 v CTRL** | | |
| Nil |  |  |

| **Upregulated set of DEGs: DHA20 (4DPT) v CTRL and Nema 4DPT/3DPI v CTRL** | | |
| --- | --- | --- |
| GO term | Function | p adjusted |
| **Common in DHA20 (4DPT) v CTRL and Nema 4DPT/3DPI v CTRL** | | |
| GO:MF | Hydrolase activity, hydrolyzing O-glycosyl compounds | 1.04×10^-6^ |
| GO:MF | Hydrolase activity, acting on glycosyl bonds | 6.09×10^-6^ |
| GO:MF | **Oxidoreductase activity** | 7.58×10^-4^ |
| GO:MF | **Abscisic acid binding** | 0.001 |
| GO:MF | **Isoprenoid binding** | 0.001 |
| GO:MF | Protein phosphatase inhibitor activity | 0.003 |
| GO:MF | **Hormone binding** | 0.003 |
| GO:MF | Phosphatase inhibitor activity | 0.003 |
| GO:MF | Alcohol binding | 0.003 |
| GO:MF | Monocarboxylic acid binding | 0.010 |
| GO:MF | **Chitinase activity** | 0.010 |
| GO:MF | **Oxidoreductase activity, acting on CH-OH group of donors** | 0.020 |
| GO:MF | Protein phosphatase regulator activity | 0.040 |
| GO:MF | Phosphatase regulator activity | 0.040 |
| GO:MF | **Peroxidase activity** | 0.040 |
| GO:MF | **Oxidoreductase activity, acting on peroxide as acceptor** | 0.040 |
| GO:MF | Manganese ion binding | 0.050 |
| GO:BP | **Defense response** | 4.21×10^-5^ |
| GO:BP | Carbohydrate metabolic process | 0.0002 |
| GO:BP | Regulation of protein serine/threonine phosphatase activity | 0.003 |
| GO:BP | **Oxidation-reduction process** | 0.006 |
| GO:BP | **Response to stress** | 0.008 |
| GO:BP | Negative regulation of hydrolase activity | 0.008 |
| GO:BP | **Cellular detoxification** | 0.010 |
| GO:BP | **Cellular response to toxic substance** | 0.010 |
| GO:BP | Negative regulation of phosphoprotein phosphatase activity | 0.010 |
| GO:BP | Negative regulation of protein dephosphorylation | 0.010 |
| GO:BP | **Detoxification** | 0.010 |
| GO:BP | Negative regulation of phosphatase activity | 0.020 |
| GO:BP | Negative regulation of dephosphorylation | 0.020 |
| GO:BP | **Response to toxic substance** | 0.010 |
| GO:BP | **Defense response to fungus** | 0.040 |
| GO:CC | Extracellular region | 1.61×10^-9^ |
| GO:CC | Anchored component of plasma membrane | 3.18×10^-4^ |
| GO:CC | Anchored component of membrane | 4.99×10^-4^ |
| GO:CC | Intrinsic component of plasma membrane | 9.36×10^-4^ |
| KEGG | **Biosynthesis of secondary metabolites** | 9.17×10^-5^ |
| KEGG | Alpha-Linolenic acid metabolism | 0.005 |
| KEGG | **Metabolic pathways** | 0.008 |
| KEGG | **Diterpenoid biosynthesis** | 0.030 |
| KEGG | Glycolysis / Gluconeogenesis | 0.040 |
|  |  |  |
| **DHA20 (4DPT) v CTRL** | | |
| GO:MF | Heme binding | 1.87×10^-8^ |
| GO:MF | Tetrapyrrole binding | 2.83×10^-8^ |
| GO:MF | **oxidoreductase activity** | 3.30×10^-6^ |
| GO:MF | Metal ion binding | 1.29×10^-4^ |
| GO:MF | Cation binding | 1.50×10^-4^ |
| GO:MF | **Oxidoreductase activity, acting on paired donors, with incorporation or reduction of molecular oxygen, NAD(P)H as one donor, and incorporation of one atom of oxygen** | 0.001 |
| GO:MF | Monooxygenase activity | 0.001 |
| GO:MF | Iron ion binding | 0.002 |
| GO:MF | **Oxidoreductase activity, acting on paired donors, with incorporation or reduction of molecular oxygen** | 0.003 |
| GO:MF | Transition metal ion binding | 0.006 |
| GO:BP | **Diterpenoid biosynthetic process** | 8.50×10^-9^ |
| GO:BP | **Diterpenoid metabolic process** | 3.36×10^-8^ |
| GO:BP | **Terpenoid biosynthetic process** | 4.32×10^-5^ |
| GO:BP | **Terpenoid metabolic process** | 1.04×10^-4^ |
| GO:BP | **Isoprenoid biosynthetic process** | 2.61×10^-4^ |
| GO:BP | **Isoprenoid metabolic process** | 6.78×10^-4^ |
| GO:BP | **Oxidation-reduction process** | 9.93×10^-4^ |
| GO:BP | **Defense response** | 0.001 |
| GO:BP | **Diterpene phytoalexin metabolic process** | 0.020 |
| GO:BP | **Diterpene phytoalexin biosynthetic process** | 0.020 |
| GO:BP | Lipid biosynthetic process | 0.020 |
| GO:BP | **Response to stress** | 0.040 |
| GO:CC | Extracellular region | 1.59×10^-6^ |
| KEGG | **Diterpenoid biosynthesis** | 1.02×10^-5^ |
| KEGG | **Biosynthesis of secondary metabolites** | 1.88×10^-4^ |
|  |  |  |
| **Nematode (4DPT/3DPI) v CTRL** | | |
| GO:MF | **Catalytic activity** | 8.05×10^-7^ |
| GO:MF | **Oxidoreductase activity** | 3.28×10^-5^ |
| GO:MF | Structural constituent of ribosome | 3.50×10^-5^ |
| GO:MF | Hydrolase activity, hydrolyzing O-glycosyl compounds | 1.81×10^-4^ |
| GO:MF | Vitamin binding | 6.49×10^-4^ |
| GO:MF | **Peroxidase activity** | 9.93×10^-4^ |
| GO:MF | **Oxidoreductase activity, acting on peroxide as acceptor** | 0.001 |
| GO:MF | Hydrolase activity, acting on glycosyl bonds | 0.001 |
| GO:MF | **Antioxidant activity** | 0.002 |
| GO:MF | Active transmembrane transporter activity | 0.003 |
| GO:MF | Structural molecule activity | 0.003 |
| GO:MF | Anion transmembrane transporter activity | 0.009 |
| GO:MF | Secondary active transmembrane transporter activity | 0.010 |
| GO:MF | **Chitinase activity** | 0.030 |
| GO:MF | Transmembrane transporter activity | 0.040 |
| GO:BP | Small molecule metabolic process | 4.26×10^-6^ |
| GO:BP | Organic acid metabolic process | 1.01×10^-5^ |
| GO:BP | **Detoxification** | 1.47×10^-4^ |
| GO:BP | Aromatic amino acid family metabolic process | 2.69×10^-4^ |
| GO:BP | **Response to toxic substance** | 2.288×10^-4^ |
| GO:BP | Oxoacid metabolic process | 3.60×10^-4^ |
| GO:BP | **Cellular detoxification** | 3.75×10^-4^ |
| GO:BP | **Cellular response to toxic substance** | 3.75×10^-4^ |
| GO:BP | **Oxidation-reduction process** | 3.77×10^-4^ |
| GO:BP | Carbohydrate metabolic process | 4.57×10^-4^ |
| GO:BP | **Hydrogen peroxide catabolic process** | 4.72×10^-4^ |
| GO:BP | **Cellular oxidant detoxification** | 5.82×10^-4^ |
| GO:BP | Carboxylic acid metabolic process | 6.00×10^-4^ |
| GO:BP | **Hydrogen peroxide metabolic process** | 8.86×10^-4^ |
| GO:BP | Aromatic amino acid family biosynthetic process | 0.001 |
| GO:BP | Carbohydrate catabolic process | 0.001 |
| GO:BP | Cellular amino acid metabolic process | 0.003 |
| GO:BP | Cellular amino acid biosynthetic process | 0.004 |
| GO:BP | Cell wall organization or biogenesis | 0.004 |
| GO:BP | **Organonitrogen compound biosynthetic process** | 0.004 |
| GO:BP | Alpha-amino acid metabolic process | 0.004 |
| GO:BP | **Reactive oxygen species metabolic process** | 0.010 |
| GO:BP | Small molecule biosynthetic process | 0.010 |
| GO:BP | Alpha-amino acid biosynthetic process | 0.020 |
| GO:BP | Organic acid biosynthetic process | 0.020 |
| GO:CC | Cytosolic ribosome | 1.92×10^-13^ |
| GO:CC | Extracellular region | 5.84×10^-12^ |
| GO:CC | Ribosomal subunit | 1.01×10^-11^ |
| GO:CC | Cytosolic small ribosomal subunit | 9.08×10^-10^ |
| GO:CC | Small ribosomal subunit | 2.10×10^-9^ |
| GO:CC | Cytosol | 4.74×10^-8^ |
| GO:CC | Ribosome | 1.52×10^-6^ |
| GO:CC | Ribonucleoprotein complex | 1.46×10^-5^ |
| GO:CC | Cell wall | 1.49×10^-4^ |
| GO:CC | External encapsulating structure | 1.75×10^-4^ |
| GO:CC | Cytosolic large ribosomal subunit | 0.003 |
| GO:CC | Cell periphery | 0.007 |
| GO:CC | Large ribosomal subunit | 0.034 |
| KEGG | Metabolic pathways | 2.64×10^-16^ |
| KEGG | Biosynthesis of amino acids | 3.72×10^-8^ |
| KEGG | **Biosynthesis of secondary metabolites** | 7.55×10^-7^ |
| KEGG | **Phenylalanine, tyrosine and tryptophan biosynthesis** | 4.03×10^-5^ |
| KEGG | Cysteine and methionine metabolism | 3.02×10^-4^ |
| KEGG | 2-Oxocarboxylic acid metabolism | 0.009 |
| KEGG | Carbon metabolism | 0.010 |
| KEGG | Arginine biosynthesis | 0.020 |
| KEGG | **Phenylpropanoid biosynthesis** | 0.020 |

| **Dodnregulated set of DEGs: DHA20 (4DPT) v CTRL and Nema 4DPT/3DPI v CTRL** | | |
| --- | --- | --- |
| **Common in DHA20 (4DPT) v CTRL and Nema 4DPT/3DPI v CTRL** | | |
| KEGG | Protein processing in endoplasmic reticulum | 0.040 |
|  |  |  |
| **DHA20 (4 DPT) v CTRL** | | |
| Nill |  |  |
|  |  |  |
| **Nematode (3DPI/4DPT) v CTRL** | | |
| GO:MF | DNA-binding transcription factor activity | 2.77×10^-7^ |
| GO:MF | Transcription regulator activity | 9.72×10^-7^ |
| GO:MF | Sequence-specific DNA binding | 1.51×10^-5^ |
| GO:MF | DNA binding | 0.002 |
| GO:MF | Trehalose-phosphatase activity | 0.010 |
| GO:MF | Water channel activity | 0.030 |
| GO:MF | Water transmembrane transporter activity | 0.030 |
| GO:BP | Regulation of RNA metabolic process | 8.10×10^-5^ |
| GO:BP | Regulation of nucleobase-containing compound metabolic process | 2.24×10^-4^ |
| GO:BP | Regulation of RNA biosynthetic process | 2.296×10^-4^ |
| GO:BP | Regulation of nucleic acid-templated transcription | 2.97×10^-4^ |
| GO:BP | Regulation of transcription, DNA-templated | 4.36×10^-4^ |
| GO:BP | Trehalose biosynthetic process | 4.97×10^-4^ |
| GO:BP | Trehalose metabolic process | 9.65×10^-4^ |
| GO:BP | Nucleic acid-templated transcription | 0.001 |
| GO:BP | **Response to oxygen-containing compound** | 0.001 |
| GO:BP | RNA biosynthetic process | 0.001 |
| GO:BP | Regulation of macromolecule biosynthetic process | 0.001 |
| GO:BP | Transcription, DNA-templated | 0.002 |
| GO:BP | **Response to water deprivation** | 0.002 |
| GO:BP | **Response to water** | 0.002 |
| GO:BP | Regulation of cellular biosynthetic process | 0.002 |
| GO:BP | Regulation of biosynthetic process | 0.003 |
| GO:BP | **Response to abiotic stimulus** | 0.003 |
| GO:BP | Regulation of cellular macromolecule biosynthetic process | 0.003 |
| GO:BP | Cytokinin-activated signaling pathway | 0.004 |
| GO:BP | **Response to acid chemical** | 0.004 |
| GO:BP | Oligosaccharide biosynthetic process | 0.005 |
| GO:BP | Cellular response to cytokinin stimulus | 0.005 |
| GO:BP | Biological regulation | 0.006 |
| GO:BP | Disaccharide biosynthetic process | 0.007 |
| GO:BP | **Response to chemical** | 0.007 |
| GO:BP | **Response to organic substance** | 0.008 |
| GO:BP | Regulation of cellular metabolic process | 0.008 |
| GO:BP | Response to inorganic substance | 0.008 |
| GO:BP | Cellular response to organic substance | 0.010 |
| GO:BP | **Regulation of gene expression** | 0.010 |
| GO:BP | **Hormone-mediated signaling pathway** | 0.010 |
| GO:BP | Phosphorelay signal transduction system | 0.010 |
| GO:BP | **Response to heat** | 0.020 |
| GO:BP | Regulation of cellular process | 0.020 |
| GO:BP | Regulation of nitrogen compound metabolic process | 0.020 |
| GO:BP | Regulation of primary metabolic process | 0.030 |
| GO:BP | **Cellular response to hormone stimulus** | 0.030 |
| GO:BP | **Cellular response to endogenous stimulus** | 0.040 |
| GO:BP | Regulation of biological process | 0.040 |
| GO:BP | Fluid transport | 0.050 |
| GO:BP | Water transport | 0.050 |
| GO:CC | Nucleus | 0.010 |
| KEGG | **Plant hormone signal transduction** | <0.001 |

| **Upregulated set of DEGs: DHA20 (4DPT) v CTRL, Nema 4DPT/3DPI v CTRL and DHA20 (4DPT/3DPI)+Nematode v CTRL** | | |
| --- | --- | --- |
| **Common in Upregulated set of DEGs: DHA20 (4DPT) v CTRL, Nema 4DPT/3DPI v CTRL and DHA20 (4DPT/3DPI)+Nematode v CTRL** | | |
| GO:MF | Hydrolase activity, hydrolyzing O-glycosyl compounds | <0.001 |
| GO:MF | Hydrolase activity, acting on glycosyl bonds | <0.001 |
| GO:MF | **Oxidoreductase activity** | <0.001 |
| GO:MF | **Abscisic acid binding** | <0.001 |
| GO:MF | **Isoprenoid binding** | <0.001 |
| GO:MF | Protein phosphatase inhibitor activity | 0.002 |
| GO:MF | **Hormone binding** | 0.002 |
| GO:MF | Phosphatase inhibitor activity | 0.002 |
| GO:MF | Alcohol binding | 0.002 |
| GO:MF | Monocarboxylic acid binding | 0.009 |
| GO:MF | Chitinase activity | 0.010 |
| GO:MF | **Oxidoreductase activity, acting on CH-OH group of donors** | 0.010 |
| GO:MF | **Catalytic activity** | 0.020 |
| GO:MF | **Peroxidase activity** | 0.020 |
| GO:MF | **Oxidoreductase activity, acting on peroxide as acceptor** | 0.020 |
| GO:MF | Protein phosphatase regulator activity | 0.020 |
| GO:MF | Phosphatase regulator activity | 0.020 |
| GO:MF | Organic acid binding | 0.030 |
| GO:MF | Enzyme inhibitor activity | 0.040 |
| GO:MF | **Oxidoreductase activity, acting on the CH-OH group of donors, NAD or NADP as acceptor** | 0.040 |
| GO:BP | **Defense response** | <0.001 |
| GO:BP | Carbohydrate metabolic process | <0.001 |
| GO:BP | Regulation of protein serine/threonine phosphatase activity | 0.002 |
| GO:BP | Obsolete oxidation-reduction process | 0.003 |
| GO:BP | Negative regulation of hydrolase activity | 0.005 |
| GO:BP | **Cellular response to toxic substance** | 0.006 |
| GO:BP | **Cellular detoxification** | 0.006 |
| GO:BP | **Detoxification** | 0.007 |
| GO:BP | **Response to toxic substance** | 0.009 |
| GO:BP | Negative regulation of protein dephosphorylation | 0.010 |
| GO:BP | Negative regulation of phosphoprotein phosphatase activity | 0.010 |
| GO:BP | Negative regulation of phosphatase activity | 0.011 |
| GO:BP | Negative regulation of dephosphorylation | 0.011 |
| GO:BP | **Response to stress** | 0.013 |
| GO:BP | **Defense response to fungus** | 0.026 |
| GO:BP | **Response to biotic stimulus** | 0.029 |
| GO:BP | **Response to fungus** | 0.040 |
| GO:BP | **Cellular response to chemical stimulus** | 0.040 |
| GO:CC | Anchored component of plasma membrane | 0.001 |
| GO:CC | Intrinsic component of plasma membrane | 0.001 |
| GO:CC | Anchored component of membrane | 0.001 |
| KEGG | **Biosynthesis of secondary metabolites** | <0.001 |
| KEGG | Alpha-Linolenic acid metabolism | 0.005 |
| KEGG | Metabolic pathways | 0.008 |
| KEGG | **Diterpenoid biosynthesis** | 0.030 |
| KEGG | Glycolysis / Gluconeogenesis | 0.042 |
|  |  |  |
|  |  |  |
| **Downregulated set of DEGs: DHA20 (4DPT) v CTRL, Nema 4DPT/3DPI v CTRL and DHA20 (4DPT/3DPI)+Nematode v CTRL** | | |
| **Common in downregulated set of DEGs: DHA20 (4DPT) v CTRL, Nema 4DPT/3DPI v CTRL and DHA20 (4DPT/3DPI)+Nematode v CTRL** | | |
| KEGG | Protein processing in endoplasmic reticulum | 0.039 |
