# Supplementary material for: Dehydroascorbate induces plant resistance in rice against root‐knot nematode Meloidogyne graminicola
Source: Mol Plant Pathol. 2022 May 19;23(9):1303–19. doi: 10.1111/mpp.13230 (PMC9366072; doi:10.1111/mpp.13230)
Supplement: Supplementary file 10 — TABLE S4 Detailed MapMan analysis showing the significantly enriched processes based on mRNA‐Seq data of 5 and 20 mM dehydroascorbate (DHA) at 1 and 4 days posttreatment (DPT), nematode‐inoculated at 3 days postinoculation (DPI) compared to mock‐treated plants, and DHA20 + nematode‐inoculated plants at 3 DPI in comparison with mock‐treated + nematode‐inoculated plants. Enriched processes highlighted in bold are mainly associated with plant stress responses. p values are FDR adjusted. CTRL, mock‐treated control plants [file MPP-23-1303-s013.docx]

**TABLE S4** Detailed Mapman analysis showing the significantly enriched processes based on mRNA-seq data of 5 and 20 mM DHA at 1 and 4 days post treatment, nematode inoculated at 3 days post inoculation compared to mock treated plants and DHA20+nematode inoculated plants at 3 days post nematode inoculation in comparison with mock treated+nematode inoculated plants. Enriched processes highlighted in bold are mainly associated with plant stress responses. P-values are FDR-adjusted. CTRL: mock-treated control plants.

| **DHA5 v CTRL (1DPT)** |  |
| --- | --- |
| **Processes** | **p value** |
| Photo synthesis | 3.16×10-7 |
| RNA | 3.16×10-7 |
| Lipid metabolism | 2.36×10-6 |
| Cell wall | 8.00×10-6 |
| **RNA.regulation of transcription** | 1.68×10-6 |
| Mitochondrial electron transport / ATP synthesis | 2.36×10-5 |
| **Misc.peroxidases** | 3.81×10-5 |
| Lipid metabolism.FA synthesis and FA elongation | 6.18×10-5 |
| Protein.degradation.ubiquitin.proteasom | 8.97×10-5 |
| Misc.gluco-, galacto- and mannosidases | 8.97×10-5 |
| **Hormone metabolism.ethylene** | 1.82×10-4 |
| Mitochondrial electron transport / ATP synthesis.NADH-DH.localisation not clear | 2.83×10-4 |
| Mitochondrial electron transport / ATP synthesis.NADH-DH | 3.33×10-4 |
| Protein.synthesis.ribosomal protein | 3.38×10-4 |
| TCA / org. transformation | 0.002 |
| Cell wall.cell wall proteins | 0.003 |
| **PS.lightreaction** | 0.003 |
| Lipid metabolism.lipid degradation.beta-oxidation | 0.003 |
| Protein.targeting.nucleus | 0.004 |
| TCA / org. transformation.TCA | 0.005 |
| **RNA.regulation of transcription.AP2/EREBP, APETALA2/Ethylene-responsive element binding protein family** | 0.005 |
| Minor CHO metabolism.trehalose | 0.005 |
| Minor CHO metabolism.others | 0.005 |
| PS.calvin cycle | 0.006 |
| RNA.regulation of transcription.C2C2(Zn) DOF zinc finger family | 0.006 |
| Protein.synthesis.ribosomal protein.prokaryotic | 0.007 |
| **hormone metabolism.ethylene.signal transduction** | 0.010 |
| Cell wall.cellulose synthesis.cellulose synthase | 0.010 |
| **PS.lightreaction.photosystem II.LHC-II** | 0.020 |
| Cell wall.modification | 0.020 |
| Cell wall.cell wall proteins.AGPs | 0.020 |
| **RNA.regulation of transcription.putative transcription regulator** | 0.020 |
| Protein.synthesis.ribosomal protein.eukaryotic.60S subunit | 0.020 |
| Cell wall.cell wall proteins.AGPs.AGP | 0.020 |
| **Redox.dismutases and catalases** | 0.020 |
| Development.unspecified | 0.020 |
| Lipid metabolism.FA synthesis and FA elongation.acyl coa ligase | 0.030 |
| **Hormone metabolism** | 0.030 |
| Lipid metabolism.lipid degradation | 0.050 |
| Signalling.receptor kinases.S-locus glycoprotein like | 0.040 |
| Redox | 0.040 |
| **Hormone metabolism.jasmonate.synthesis-degradation** | 0.040 |
| **Hormone metabolism.ethylene.synthesis-degradation** | 0.040 |
| **PS.lightreaction.photosystem II** | 0.050 |
| Development | 0.050 |
| Cell wall.cellulose synthesis | 0.050 |
| **Hormone metabolism.jasmonate** | 0.050 |
| Protein.synthesis.ribosomal protein.prokaryotic.unknown organellar | 0.050 |

| **DHA5 v CTRL (4DPT)** |  |
| --- | --- |
| **Processes** | **p value** |
| Cell wall.cell wall proteins.AGPs | 2.3×10-5 |
| Cell wall.cell wall proteins.AGPs.AGP | 2.38×10-5 |
| Cell wall | 6.69×10-5 |
| Protein.degradation.ubiquitin.E3 | 6.69×10-5 |
| Transport.Major Intrinsic Proteins.PIP | 7.58×10-4 |
| Misc.gluco-, galacto- and mannosidases.endoglucanase | 0.001 |
| Amino acid metabolism.synthesis.aromatic aa | 0.001 |
| **Stress.abiotic.heat** | 0.001 |
| Misc.UDP glucosyl and glucoronyl transferases | 0.003 |
| Protein.degradation.ubiquitin.E3.RING | 0.004 |
| **Hormone metabolism.gibberelin.induced-regulated-responsive-activated** | 0.005 |
| Amino acid metabolism.degradation.branched chain group | 0.006 |
| Transport.Major Intrinsic Proteins | 0.007 |
| Misc | 0.020 |
| Cell wall.cellulose synthesis.cellulose synthase | 0.020 |
| **Signalling.phosphinositides** | 0.020 |
| Signalling.receptor kinases.S-locus glycoprotein like | 0.030 |
| Signalling.phosphinositides.inositol-1,3,4-trisphosphate 5/6-kinase | 0.030 |
| Amino acid metabolism.degradation.branched-chain group.shared | 0.030 |
| Cell wall.modification | 0.030 |
| Amino acid metabolism.synthesis.aromatic aa.tryptophan.tryptophan synthase | 0.030 |
| Glycolysis | 0.040 |
| Protein | 0.040 |
| Lipid metabolism.lipid degradation | 0.040 |
| Protein.synthesis.initiation | 0.050 |
| Protein.degradation.ubiquitin.E3.SCF.FBOX | 0.050 |
| Protein.targeting | 0.050 |
| Protein.degradation.ubiquitin | 0.050 |

| **DHA20 v CTRL (1DPT)** |  |
| --- | --- |
| **Processes** | **p value** |
| Protein.synthesis.ribosomal protein.eukaryotic.40S subunit | 4.48×10-10 |
| Protein.synthesis.ribosomal protein | 2.52×10-9 |
| Protein.synthesis.ribosomal protein.eukaryotic.60S subunit | 3.55×10-9 |
| **PS.lightreaction** | 1.04×10-8 |
| Protein.synthesis | 6.90×10-7 |
| Amino acid metabolism.synthesis | 1.14×10-7 |
| Amino acid metabolism | 7.51×10-7 |
| **PS** | 8.87×10-7 |
| **Secondary metabolism** | 1.01×10-6 |
| Amino acid metabolism.synthesis.aromatic aa | 1.84×10-6 |
| Signalling.receptor kinases | 5.15×10-6 |
| **Secondary metabolism.phenylpropanoids** | 6.57×10-6 |
| **PS.lightreaction.photosystem II** | 1.04×10-5 |
| Protein.postranslational modification.kinase.receptor like cytoplasmatic kinase VII | 1.22×10-5 |
| Amino acid metabolism.synthesis.aromatic aa.tryptophan | 1.41×10-5 |
| Protein.postranslational modification.kinase | 2.33×10-5 |
| TCA / org. transformation.TCA | 2.81×10-5 |
| **Secondary metabolism.phenylpropanoids.lignin biosynthesis** | 5.02×10-5 |
| Protein.degradation.ubiquitin.proteasom | 7.32×10-5 |
| Signalling.receptor kinases.S-locus glycoprotein like | 7.93×10-5 |
| **PS.lightreaction.photosystem II.LHC-II** | 1.00×10-4 |
| Protein.degradation.ubiquitin.E3 | 1.08×10-4 |
| Protein | 1.16×10-4 |
| Mitochondrial electron transport / ATP synthesis | 2.55×10-4 |
| Transport | 4.86×10-4 |
| Signalling | 4.86×10-4 |
| Signalling.receptor kinases.misc | 5.62×10-4 |
| Misc.beta 1,3 glucan hydrolases.glucan endo-1,3-beta-glucosidase | 7.76×10-4 |
| Protein.degradation.ubiquitin.E3.SCF.FBOX | 0.001 |
| TCA / org. transformation | 0.001 |
| Transport.misc | 0.002 |
| Protein.degradation.ubiquitin.E3.SCF | 0.002 |
| RNA | 0.002 |
| Misc.beta 1,3 glucan hydrolases | 0.003 |
| **Hormone metabolism.jasmonate.synthesis-degradation** | 0.003 |
| **PS.lightreaction.photosystem I.PSI polypeptide subunits** | 0.003 |
| **Hormone metabolism.jasmonate** | 0.004 |
| **RNA.regulation of transcription.WRKY domain transcription factor family** | 0.004 |
| **Secondary metabolism.isoprenoids.non-mevalonate pathway** | 0.004 |
| **PS.lightreaction.photosystem I** | 0.004 |
| **Hormone metabolism** | 0.004 |
| **RNA.regulation of transcription.MYB domain transcription factor family** | 0.006 |
| **Secondary metabolism.phenylpropanoids.lignin biosynthesis.PAL** | 0.010 |
| Nucleotide metabolism | 0.010 |
| **Hormone metabolism.ethylene** | 0.010 |
| Amino acid metabolism.synthesis.aromatic aa.tryptophan.tryptophan synthase | 0.010 |
| **Protein.targeting.secretory pathway.ER** | 0.010 |
| RNA.regulation of transcription.PHOR1 | 0.020 |
| **Hormone metabolism.ethylene.synthesis-degradation** | 0.020 |
| Protein.targeting | 0.020 |
| **Amino acid metabolism.synthesis.aromatic aa.chorismate** | 0.020 |
| TCA / org. transformation.TCA.pyruvate DH | 0.020 |
| Protein.targeting.secretory pathway | 0.030 |
| Glycolysis | 0.030 |
| RNA.regulation of transcription.Psudo ARR transcription factor family | 0.030 |
| Cell.vesicle transport | 0.030 |
| **Redox** | 0.040 |
| **Secondary metabolism.isoprenoids** | 0.040 |
| Misc | 0.040 |
| Glycolysis.cytosolic branch | 0.040 |
| Cell wall.precursor synthesis | 0.040 |
| Glycolysis.cytosolic branch.enolase | 0.040 |
| Amino acid metabolism.synthesis.aromatic aa.tryptophan.anthranilate synthase | 0.040 |
| **PS.lightreaction.photosystem II.PSII polypeptide subunits** | 0.040 |
| TCA / org. transformation.TCA.pyruvate DH.E2 | 0.050 |
| Amino acid metabolism.synthesis.serine-glycine-cysteine group.serine | 0.050 |
| Nucleotide metabolism.phosphotransfer and pyrophosphatases | 0.050 |
| **Hormone metabolism.jasmonate.synthesis-degradation.lipoxygenase** | 0.050 |
| RNA.regulation of transcription.PWWP domain protein | 0.050 |
| Amino acid metabolism.synthesis.central amino acid metabolism | 0.050 |
| Signalling.calcium | 0.050 |
| **Transport.hormones** | 0.060 |
| **Transport.hormones.auxin** | 0.060 |

| **DHA20 v CTRL (4DPT)** |  |
| --- | --- |
| **Processes** | **p value** |
| RNA | 1.90×10-16 |
| **RNA.regulation of transcription** | 7.41×10-14 |
| Protein.synthesis.ribosomal protein.eukaryotic.40S subunit | 1.98×10-12 |
| Protein | 1.78×10-7 |
| Amino acid metabolism.synthesis | 1.78×10-7 |
| Protein.targeting | 1.74×10-7 |
| Amino acid metabolism | 2.38×10-7 |
| **Misc.peroxidases** | 1.78×10-6 |
| Transport | 4.38×10-6 |
| Protein.degradation.ubiquitin.proteasom | 2.27×10-5 |
| Protein.synthesis.initiation | 3.08×10-5 |
| Protein.degradation.ubiquitin.E3.RING | 1.81×10-4 |
| Protein.degradation.ubiquitin.E3 | 2.65×10-4 |
| Misc.glutathione S transferases | 4.13×10-4 |
| Protein.targeting.secretory pathway | 4.27×10-4 |
| Mitochondrial electron transport / ATP synthesis | 4.35×10-4 |
| Not assigned | 4.35×10-4 |
| Not assigned.unknown | 4.35×10-4 |
| Cell wall | 8.20×10-4 |
| Misc.UDP glucosyl and glucoronyl transferases | 0.001 |
| **Stress.biotic.PR-proteins** | 0.001 |
| Protein.targeting.secretory pathway.ER | 0.002 |
| **RNA.regulation of transcription.HSF,Heat-shock transcription factor family** | 0.003 |
| **Signalling.phosphinositides** | 0.004 |
| RNA.processing.splicing | 0.004 |
| Amino acid metabolism.synthesis.glutamate family | 0.006 |
| Fermentation | 0.006 |
| **Secondary metabolism** | 0.008 |
| Cell wall.degradation | 0.009 |
| **Redox.ascorbate and glutathione** | 0.009 |
| Misc.GDSL-motif lipase | 0.009 |
| Protein.folding | 0.010 |
| Misc.gluco-, galacto- and mannosidases.alpha-galactosidase | 0.010 |
| Transport.p- and v-ATPases.H+-transporting two-sector ATPase | 0.010 |
| Minor CHO metabolism.trehalose.TPS | 0.010 |
| Amino acid metabolism.synthesis.central amino acid metabolism.aspartate | 0.010 |
| Amino acid metabolism.synthesis.central amino acid Metabolism.aspartate.aspartate aminotransferase | 0.010 |
| **Redox** | 0.010 |
| Signalling.receptor kinases.S-locus glycoprotein like | 0.010 |
| Rna.regulation of transcription.MYB domain transcription factor family | 0.020 |
| Lipid metabolism.''exotics'' (steroids, squalene etc).sphingolipids | 0.020 |
| Transport.peptides and oligopeptides | 0.020 |
| Amino acid metabolism.synthesis.glutamate family.arginine | 0.020 |
| Amino acid metabolism.synthesis.serine-glycine-cysteine group.cysteine.OASTL | 0.020 |
| Transport.metabolite transporters at the envelope membrane | 0.020 |
| Minor CHO metabolism.trehalose.potential TPS/TPP | 0.030 |
| Signalling.phosphinositides.inositol-1,3,4-trisphosphate 5/6-kinase | 0.030 |
| Misc.nitrilases, *nitrile lyases, berberine bridge enzymes, reticuline oxidases, troponine reductases | 0.030 |
| Fermentation.PDC | 0.040 |
| **RNA.regulation of transcription.putative transcription regulator** | 0.040 |
| Misc.gluco-, galacto- and mannosidases | 0.040 |
| RNA.regulation of transcription.Chromatin Remodeling Factors | 0.040 |
| RNA.regulation of transcription.MYB-related transcription factor family | 0.040 |
| Lipid metabolism.''exotics'' (steroids, squalene etc) | 0.040 |
| Major CHO metabolism.degradation.sucrose | 0.040 |
| Nucleotide metabolism.phosphotransfer and pyrophosphatases.nucleoside diphosphate kinase | 0.040 |
| Lipid metabolism.lipid degradation | 0.050 |
| **Stress.abiotic.heat** | 0.050 |
| **N-metabolism.misc** | 0.060 |

| **Nematode v CTRL (4DPT/3DPI)** |  |
| --- | --- |
| **Processes** | **p value** |
| Protein.synthesis.ribosomal protein.eukaryotic.40S subunit | 4.34×10-14 |
| Protein | 1.48×10-13 |
| Amino acid metabolism.synthesis | 7.94×10-11 |
| Amino acid metabolism | 7.94×10-11 |
| Protein.degradation.ubiquitin.proteasom | 1.06×10-7 |
| Mitochondrial electron transport / ATP synthesis | 6.29×10-7 |
| Misc.UDP glucosyl and glucoronyl transferases | 1.93×10-6 |
| Protein.targeting | 3.03×10-6 |
| Not assigned | 4.40×10-6 |
| Not assigned.unknown | 4.40×10-6 |
| Protein.synthesis.initiation | 1.21×10-5 |
| Transport | 2.23×10-5 |
| RNA | 3.99×10-5 |
| **RNA.regulation of transcription** | 9.10×10-5 |
| Cell wall | 1.82×10-4 |
| Protein.degradation.ubiquitin.E3 | 3.56×10-4 |
| Protein.targeting.mitochondria | 3.87×10-4 |
| Transport.major Intrinsic Proteins.PIP | 6.94×10-4 |
| RNA.processing.splicing | 6.94×10-4 |
| **Misc.peroxidases** | 7.19×10-4 |
| Amino acid metabolism.synthesis.glutamate family | 7.19×10-4 |
| **Stress.biotic.PR-proteins** | 7.25×10-4 |
| **RNA.regulation of transcription.HSF,Heat-shock transcription factor family** | 0.001 |
| Protein.degradation.ubiquitin.E3.RING | 0.001 |
| Protein.aa activation | 0.002 |
| RNA.regulation of transcription.ARR | 0.002 |
| **PS.lightreaction.photosystem II.LHC-II** | 0.002 |
| Minor CHO metabolism.trehalose.TPS | 0.004 |
| Misc.gluco-, galacto- and mannosidases | 0.004 |
| Signalling.receptor kinases | 0.005 |
| RNA.regulation of transcription.MYB-related transcription factor family | 0.006 |
| Amino acid metabolism.synthesis.glutamate family.arginine | 0.007 |
| **PS.lightreaction** | 0.008 |
| Misc.beta 1,3 glucan hydrolases | 0.008 |
| TCA / org. transformation.TCA | 0.009 |
| Signalling.receptor kinases.S-locus glycoprotein like | 0.010 |
| Cell wall.degradation.pectate lyases and polygalacturonases | 0.010 |
| Protein.targeting.secretory pathway | 0.010 |
| Cell wall.degradation | 0.010 |
| Minor CHO metabolism.trehalose | 0.010 |
| **Signalling.phosphinositides** | 0.010 |
| **PS.lightreaction.photosystem II** | 0.010 |
| Minor CHO metabolism.trehalose.potential TPS/TPP | 0.020 |
| Nucleotide metabolism.synthesis.purine | 0.020 |
| Transport.metabolite transporters at the mitochondrial membrane | 0.020 |
| Amino acid metabolism.synthesis.serine-glycine-cysteine group | 0.020 |
| Misc.gluco-, galacto- and mannosidases.alpha-galactosidase | 0.020 |
| Amino acid metabolism.synthesis.central amino acid metabolism.aspartate | 0.020 |
| Fermentation.PDC | 0.020 |
| Amino acid metabolism.synthesis.central amino acid metabolism.aspartate.aspartate aminotransferase | 0.020 |
| **Secondary metabolism.flavonoids.flavonols** | 0.020 |
| Transport.peptides and oligopeptides | 0.030 |
| Protein.glycosylation | 0.030 |
| Lipid metabolism.FA synthesis and FA elongation.pyruvate kinase | 0.030 |
| Fermentation | 0.030 |
| Signalling.receptor kinases.leucine rich repeat III | 0.030 |
| Amino acid metabolism.synthesis.aspartate family | 0.040 |
| Signalling.calcium | 0.040 |
| Amino acid metabolism.synthesis.aromatic aa | 0.040 |
| Misc.nitrilases, *nitrile lyases, berberine bridge enzymes, reticuline oxidases, troponine reductases | 0.040 |
| Protein.synthesis.elongation | 0.040 |
| **Secondary metabolism** | 0.040 |
| Protein.targeting.secretory pathway.ER | 0.050 |
| Protein.synthesis.ribosomal protein.eukaryotic.60S subunit.P0 | 0.050 |
| Misc.gluco-, galacto- and mannosidases.endoglucanase | 0.050 |
| **Hormone metabolism.ethylene.synthesis-degradation** | 0.060 |

| **DHA20+Nematode v CTRL (4DPT/3DPI)** |  |
| --- | --- |
| **Processes** | **p value** |
| Amino acid metabolism.synthesis | 1.17×10-8 |
| Misc | 6.59×10-8 |
| Protein | 9.61×10-8 |
| Amino acid metabolism | 2.15×10-6 |
| Protein.degradation.ubiquitin.E3 | 2.67×10-6 |
| Protein.degradation.ubiquitin.E3.RING | 9.20×10-6 |
| **RNA.regulation of transcription** | 1.82×10-5 |
| Protein.targeting | 4.95×10-5 |
| Protein.degradation.ubiquitin.proteasom | 5.16×10-5 |
| RNA | 3.40×10-4 |
| Protein.synthesis.initiation | 4.58×10-4 |
| Protein.synthesis.ribosomal protein.prokaryotic | 6.68×10-4 |
| Not assigned | 6.68×10-4 |
| Not assigned.unknown | 6.68×10-4 |
| Protein.synthesis.elongation | 7.28×10-4 |
| Protein.folding | 0.015 |
| Transport.Major Intrinsic Proteins.PIP | 0.015 |
| Misc.UDP glucosyl and glucoronyl transferases | 0.002 |
| **RNA.regulation of transcription.HSF,Heat-shock transcription factor family** | 0.002 |
| Lipid metabolism.lipid degradation | 0.002 |
| RNA.processing.splicing | 0.002 |
| Amino acid metabolism.synthesis.glutamate family | 0.008 |
| Misc.gluco-, galacto- and mannosidases | 0.010 |
| Minor CHO metabolism.trehalose.TPS | 0.010 |
| Protein.targeting.mitochondria | 0.010 |
| Misc.beta 1,3 glucan hydrolases | 0.010 |
| Signalling.phosphinositides | 0.010 |
| Lipid metabolism.lipid degradation.lipases | 0.020 |
| Protein.degradation.ubiquitin | 0.020 |
| Nucleotide metabolism.synthesis.purine | 0.020 |
| Transport | 0.020 |
| Amino acid metabolism.synthesis.central amino acid metabolism.aspartate | 0.020 |
| Protein.aa activation | 0.020 |
| Fermentation.PDC | 0.020 |
| Amino acid metabolism.synthesis.central amino acid metabolism.aspartate.aspartate aminotransferase | 0.020 |
| Lipid metabolism | 0.020 |
| Protein.targeting.nucleus | 0.020 |
| Protein.postranslational modification | 0.020 |
| Protein.synthesis.ribosomal protein.eukaryotic.60S subunit.P0 | 0.030 |
| Misc.gluco-, galacto- and mannosidases.alpha-galactosidase | 0.030 |
| Transport.metabolite transporters at the envelope membrane | 0.040 |
| RNA.regulation of transcription.MYB-related transcription factor family | 0.040 |
| Fermentation | 0.040 |

| **DHA20+Nematode v CTRL+Nematode (4DPT/3DPI)** |  |
| --- | --- |
| **Processes** | **p value** |
| Mitochondrial electron transport / ATP synthesis | 3.08×10-10 |
| Amino acid metabolism | 3.08×10-10 |
| **DNA.synthesis/chromatin structure.histone** | 5.33×10-10 |
| Amino acid metabolism.synthesis | 6.02×10-6 |
| Protein | 4.12×10-5 |
| Not assigned | 5.21×10-5 |
| Not assigned.unknown | 5.21×10-5 |
| DNA | 8.98×10-5 |
| Protein.degradation.ubiquitin.proteasom | 9.32×10-5 |
| DNA.synthesis/chromatin structure | 9.80×10-5 |
| Amino acid metabolism.degradation.branched chain group | 1.72×10-4 |
| Misc | 2.19×10-4 |
| RNA | 2.46×10-4 |
| **Hormone metabolism.ethylene.synthesis-degradation** | 2.78×10-4 |
| Transport | 3.90×10-4 |
| **Secondary metabolism.phenylpropanoids** | 4.70×10-4 |
| TCA / org. transformation.TCA | 7.56×10-4 |
| **Secondary metabolism** | 7.73×10-4 |
| Protein.synthesis.ribosomal protein.eukaryotic | 7.85×10-4 |
| Transport.p- and v-ATPases.H+-transporting two-sector ATPase | 0.001 |
| **RNA.regulation of transcription.WRKY domain transcription factor family** | 0.001 |
| Signalling.receptor kinases.S-locus glycoprotein like | 0.001 |
| Amino acid metabolism.degradation | 0.002 |
| RNA.regulation of transcription.putative transcription regulator | 0.002 |
| Amino acid metabolism.synthesis.aromatic aa.chorismate | 0.003 |
| RNA.regulation of transcription.ARR | 0.004 |
| Transport.amino acids | 0.005 |
| TCA / org. transformation | 0.005 |
| **Hormone metabolism.ethylene** | 0.006 |
| Transport.p- and v-ATPases | 0.007 |
| RNA.processing.splicing | 0.009 |
| **Stress.abiotic.heat** | 0.009 |
| Mitochondrial electron transport / ATP synthesis.cytochrome c reductase | 0.009 |
| Transport.Major Intrinsic Proteins.PIP | 0.010 |
| Amino acid metabolism.synthesis.aromatic aa | 0.010 |
| Amino acid metabolism.degradation.branched-chain group.leucine | 0.010 |
| **Hormone metabolism.jasmonate.synthesis-degradation** | 0.010 |
| **Redox.ascorbate and glutathione** | 0.020 |
| Mitochondrial electron transport / ATP synthesis.F1-ATPase | 0.020 |
| **Hormone metabolism.jasmonate** | 0.020 |
| Protein.targeting.secretory pathway | 0.020 |
| Signalling.receptor kinases | 0.020 |
| **Secondary metabolism.phenylpropanoids.lignin biosynthesis** | 0.020 |
| Amino acid metabolism.synthesis.glutamate family.arginine | 0.020 |
| Mitochondrial electron transport / ATP synthesis.NADH-DH.type II | 0.020 |
| **Hormone metabolism.gibberelin.induced-regulated-responsive-activated** | 0.020 |
| RNA.processing | 0.030 |
| **Transport.nitrate** | 0.030 |
| Protein.synthesis.ribosomal protein.eukaryotic.60S subunit | 0.030 |
| Signalling.receptor kinases.leucine rich repeat VIII-1 | 0.030 |
| Signalling.receptor kinases.leucine rich repeat VIII-2 | 0.030 |
| Redox | 0.030 |
| Protein.synthesis.ribosomal protein.prokaryotic.chloroplast.30S subunit | 0.030 |
| PS.calvin cycle.aldolase | 0.030 |
| Amino acid metabolism.synthesis.glutamate family | 0.030 |
| Protein.synthesis | 0.030 |
| Cell.organisation | 0.030 |
| Minor CHO metabolism.trehalose.potential TPS/TPP | 0.040 |
| Misc.nitrilases, *nitrile lyases, berberine bridge enzymes, reticuline oxidases, troponine reductases | 0.030 |
| Protein.degradation | 0.040 |
| **Stress.abiotic.unspecified** | 0.040 |
| **Secondary metabolism.flavonoids** | 0.040 |
| **Hormone metabolism.jasmonate.synthesis-degradation.allene oxidase synthase** | 0.040 |
| **Misc.peroxidases** | 0.040 |
| Co-factor and vitamine metabolism.pantothenate | 0.050 |
| Protein.targeting.secretory pathway.unspecified | 0.050 |
| Amino acid metabolism.synthesis.central amino acid metabolism | 0.050 |
| **RNA.regulation of transcription** | 0.050 |
| Protein.synthesis.ribosomal protein.prokaryotic.chloroplast | 0.050 |
| Lipid metabolism.FA synthesis and FA elongation.acyl coa ligase | 0.050 |
| Protein.synthesis.ribosomal protein.eukaryotic.40S subunit | 0.060 |
| Mitochondrial electron transport / ATP synthesis.cytochrome c oxidase | 0.060 |
| Amino acid metabolism.degradation.branched-chain group.shared | 0.060 |
| **Secondary metabolism.phenylpropanoids.lignin biosynthesis.PAL** | 0.060 |
